# Substrate influences human removal of freshwater turtle nests in the eastern Brazilian Amazon

**DOI:** 10.1038/s41598-020-65074-1

**Published:** 2020-05-15

**Authors:** Fernanda Michalski, Darren Norris, Itxaso Quintana, Andressa Valerio, James P. Gibbs

**Affiliations:** 1 Postgraduate Programme in Tropical Biodiversity, Federal University of Amapá, Macapá Amapá, Brazil; 2 Ecology and Conservation of Amazonian Vertebrates Research Group, Federal University of Amapá, Macapá Amapá, Brazil; 3 Pro-Carnivores Institute, Atibaia São Paulo, Brazil; 4 School of Environmental Sciences, Federal University of Amapá, Macapá Amapá, Brazil; 5 Ecology Department, Federal University of Rio Grande do Sul, Porto Alegre Rio Grande do Sul, Brazil; 6 Department of Forest and Environmental Biology, State University of New York, College of Environmental Science and Forestry, Syracuse, NY USA

**Keywords:** Ecology, Conservation biology, Freshwater ecology, Restoration ecology, Conservation biology, Freshwater ecology, Restoration ecology

## Abstract

Substrate type determines nesting success and fitness in turtles and is a critical consideration for nesting area protection and restoration. Here, we evaluated the effect of substrate on nest removal by humans in the eastern Brazilian Amazon. We analyzed substrate composition and fate of 216 *P. unifilis* nests along 88 km of rivers. River segment and substrate type were the most important predictors of nest removal by humans. We found up to 36% lower removal of nests in fine sand and experimental results support the hypothesis that wind more often obscures tracks of nesting females in substrates with more (>66%) fine sand. Our findings are useful for informing the restoration of artificial nesting areas across the Amazon, as nesting area substrates should be selected not only to maintain hatchling sex ratios, but also to minimize nest removal by humans.

## Introduction

Turtles are experiencing global population declines^1^, with approximately 61% of all species threatened with extinction or already extinct^2^. This issue is particularly acute in the tropics, where turtles represent food and income for local populations^1,3–5^. Survival of exploited turtle populations can be enhanced by habitat restoration^6–8^. For example, increasing available nesting habitat improved population growth in threatened marine turtles^9,10^ and recruitment in temperate freshwater turtles^11,12^. In contrast, the restoration of terrestrial nesting habitats for tropical freshwater turtles remains poorly explored.

The Amazon basin encapsulates 21^st^ century conservation challenges for tropical freshwater turtles. Myriad threats to species and populations mean that the priorities for freshwater turtle conservation actions remain intensely debated^13^. Among the factors affecting long-term turtle population viability, adult survival has been demonstrated to be crucial^14,15^, but nest-site selection by female turtles is also a key process for reproductive success and maternal survival^16–18^. The once common freshwater turtles (*Podocnemis* spp.) are declining across Amazonia due to rampant overexploitation of both adults and eggs^19,20^, and the loss and degradation of aquatic (feeding, dispersal and reproduction) and terrestrial (nesting) habitat due to deforestation^21^ and hydroelectric expansion^19^. Additionally, as with many wildlife species across Amazonia, the lack of effective enforcement of existing regulations^22,23^ and ineffective environmental impact assessments^22,24,25^ mean that conservation actions have failed to generate widespread recoveries^5,26^.

Currently, the yellow-spotted river turtle (*Podocnemis unifilis*) is classified as Vulnerable (A1acd) by the IUCN^27^, with a recommended revision to Endangered^5^. *P. unifilis* is a widespread freshwater species found across the Amazon, North Atlantic and Orinoco river basins^27,28^. This is a relatively large species (females can weight up to 12 kg) that has been exploited since the pre-colonial period (pre 18th century) and is still widely consumed by indigenous and riverine peoples across Amazonia^4,20,29^. Continued overexploitation means that egg harvest by humans is one of the main causes of population reductions throughout its range^30,31^. Brazil covers 57% of the species range^28^ and is therefore a vital focus for the conservation of *P. unifilis*. Yet, the drastic reductions in science funding in Brazil^32,33^, means that any conservation efforts must be strategic and focused within the management actions that are most likely to succeed in terms of turtle and biodiversity conservation.

New dams present a unique challenge to freshwater turtles in the area of reservoir formations due to riverbank habitat alteration and irreversible submersion of nesting areas used by these species^22,25^. It is therefore critical to have information to restore nesting areas to mitigate the negative effects of hydropower development on Amazonian freshwater turtles. Although egg survival produces a relatively small overall effect on turtle population growth rates when compared to adult survival^15^, increasing survival of eggs and hatchlings can compensate for decreases in adult survival in *P. unifilis*^28^. Considering the importance of nest-site selection, the restoration of nesting habitat could be expected to increase *P. unifilis* populations, however, there remains a lack of information (including the nature of nesting substrates associated with nesting success) to effectively develop restoration actions.

Nesting patterns associated with environmental variables have been widely documented for freshwater turtle species, including for *P. unifilis*^30,34,35^. Most of the available *P. unifilis* nesting literature focuses on describing physical characteristics of nesting areas and temperature effects on incubation period^36,37^ and sex ratio determination^38^. A recent study suggests that the principal substrate type is also an important factor explaining the proportion of *P. unifilis* nests removed by humans^30^. Yet, to our knowledge, there are no studies evaluating the effects of substrate type on predators of *P. unifilis* nests. Most nest predators search and find turtle nests largely through scent^17^ whereas humans, which have rapidly become the dominant predator of freshwater turtle nests across the Amazon basin^20,31^, find nests by visually following the nesting females or their characteristic tracks^20,31^. Substrate type is known to influence the conspicuousness and persistence of turtle tracks^39^. It is therefore to be expected that substrate type may influence the ability of humans to detect *P. unifilis* nest-sites.

Our goal was to provide evidence to inform best practice for nesting area restoration to maximize reproductive success in *P. unifilis*. Here, we predict that *P. unifilis* nest-sites with different substrate types will have different removal rates by humans. To test this prediction we evaluated substrate of 216 *P. unifilis* nests on 43 nesting areas along 88 km of river segments in the eastern Brazilian Amazon and compared their fates including removal by humans. We also examined grain size of substrate particles found in *P. unifilis* nests to identify substrate associations with nest removal by humans and guide restoration of nesting areas after dam establishment across the Amazon.

## Results

A total of 216 nests (95 in the Falsino and 121 in the Araguari river) were found in 43 nesting areas (Table 1), yielding an average of 5.02 nests per nesting area (average ± SD = 5.02 ± 3.90, range = 1–17 nests) in both river segments. More than half (n = 127, 58.80%) of all nests were removed by humans, with the highest proportion of nest removal (75.21%) in the Araguari segment (Table 1).

**Table 1:** Nesting areas encountered along the Falsino and Araguari rivers.

River segment	River length (km)	Nesting areas (count)	Nests (count)	Nests removed by humans (%)
Araguari	46	21	121	91 (75.21%)
Falsino	42	22	95	36 (37.89%)
Overall	88	43	216	127 (58.80%)

**Figure 1:**
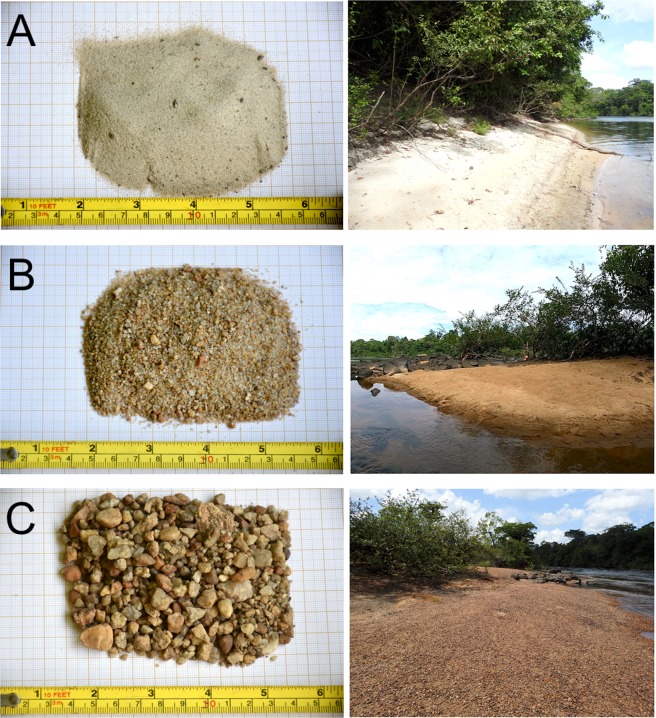
Examples of grain size particle samples obtained from *Podocnemis unifilis* nests in the eastern Brazilian Amazon. Photos show representative nesting areas and substrate from (**A**) sample composed mostly of fine sand, (**B**) sample composed of medium sand with presence of gravel, and (**C**) sample composed mostly of gravel. Photo credits: Fernanda Michalski.

The grain size distribution of nest substrates varied greatly, from nests laid in 76% fine sand to 93% gravel (Table 2, Figs. 1 and S1). However, the majority of nests were laid in substrates with higher proportions (> 50%) of sand (Table 2, Table S1). Although there was variation in substrate grain sizes between nests, there was no evidence to suggest that grain sizes differed between the Araguari and Falsino river segments (Fig. S1).

**Table 2:** Summary of grain particle sizes of *Podocnemis unifilis* nests.

Substrate grain type	River segment		Overall
	Araguari	Falsino	
Gravel (> 2 mm)	13.76 ± 18.52 (0 – 73.13)	8.86 ± 18.48 (0 – 93.26)	11.61 ± 18.66 (0 – 93.26)
Very Coarse sand (1–2 mm)	11.89 ± 12.18 (0 – 53.37)	12.05 ± 16.51 (0.01 – 66.08)	11.96 ± 14.25 (0 – 66.08)
Coarse sand (0.5–1 mm)	17.37 ± 19.44 (0.05 – 74.36)	22.72 ± 20.53 (0.09 – 72.06)	19.72 ± 20.10 (0.05 – 74.36)
Medium sand (0.25–0.5 mm)	22.79 ± 17.33 (1.73 – 77.20)	31.71 ± 21.26 (0.70 – 75.49)	26.71 ± 19.66 (0.70 – 77.20)
Fine sand (0.125–0.25 mm)	27.53 ± 23.72 (0.13 – 75.70)	20.62 ± 21.30 (0.31 – 71.50)	24.49 ± 22.95 (0.13 – 75.70)
Very fine sand (0.063–0.25 mm)	6.37 ± 8.22 (0.04 – 31.49)	3.87 ± 4.94 (0.08 – 20.55)	5.27 ± 7.08 (0.04 – 31.49)
Coarse silt (<0.063 mm)	0.30 ± 0.35 (0.01 – 1.55)	0.17 ± 0.18 (0.01 – 0.86)	0.24 ± 0.30 (0.01 – 1.55)

Values are percentages (mean ± SD, range in parentheses) obtained by dry-sieving substrate obtained from 216 *Podocnemis unifilis* nests in the eastern Brazilian Amazon.

**Figure 2:**
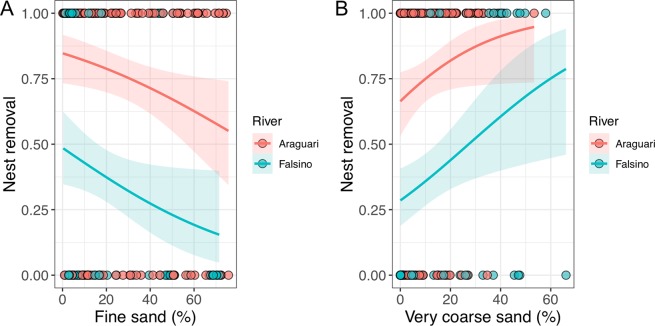
Relationship between (**A**) proportion of fine sand, and (**B**) proportion of very coarse sand, and nest removal by humans (binary variable) in 216 nests sampled within 43 nesting areas along Araguari (red) and Falsino (green) rivers. Lines (mean) and shaded areas (±95% CI) show predictions obtained from Generalized Linear Model regression (GLM).

Of the eight environmental, anthropogenic and spatial variables considered, nest removal by humans was affected (*P* < 0.05) by river segment and substrate type (Table 3). Turtle nests in the Falsino segment were removed less by humans when compared to nests located in the Araguari segment (Table 3). Marginal predictions from the GAM (where effects of other variables were controlled using mean values of continuous variables and factor reference levels) showed an average threefold difference (28.4% to 88.9%) in removal across the different substrates (range of PC1 values). Indeed, nests were removed less by humans when located on substrates with higher proportions of fine sand, the substrate type generally represented by our first PCA axis (Table 3, Fig. 2). This pattern of reduced removal with increasing proportions of fine sand was consistent across both river sections, with predicted removal declining in parallel (Fig. 2).

**Table 3:** Numerical outputs (parametric coefficients and approximate significance of smooth terms) of the Generalized Additive Model used to predict removal by humans in 216 nests, at 43 nesting areas in the eastern Brazilian Amazon.

Nominal variables	Parametric coefficients			
	Estimate	Standard error	*t*-value	*P* value^#^
Intercept	0.768	0.866	0.887	0.375
Type (Island vs bank)	1.031	0.911	1.132	0.258
River (Falsino vs Araguari)	−2.201	0.941	−2.339	0.019^*^
Distance to house	−0.671	0.499	−1.346	0.178
Nest density	−0.114	0.354	−0.321	0.748
Distance to water	0.441	0.252	1.751	0.080^+^
PC1 scores	0.422	0.196	2.152	0.031^*^
**Smoothers**	**Approximate significance of smooth terms**			
	**edf**^**s**^	**Ref.df**^**s**^	**Chi Square**	***P*** **value**^**#**^
s(Long, Lat)	7.622e-05	29	0.0	1
s(Nesting area ID)	2.346e+01	38	51.5	7.26e-05^***^
Observations	216			
Deviance explained	50.1%			

^s^edf: estimated degree of freedom; Ref.df: estimated degree of freedom for reference.

^#^Significance code: ^+^ <0.10, ^*^ <0.05, ^**^ <0.01, ^***^ <0.001.

**Figure 3:**
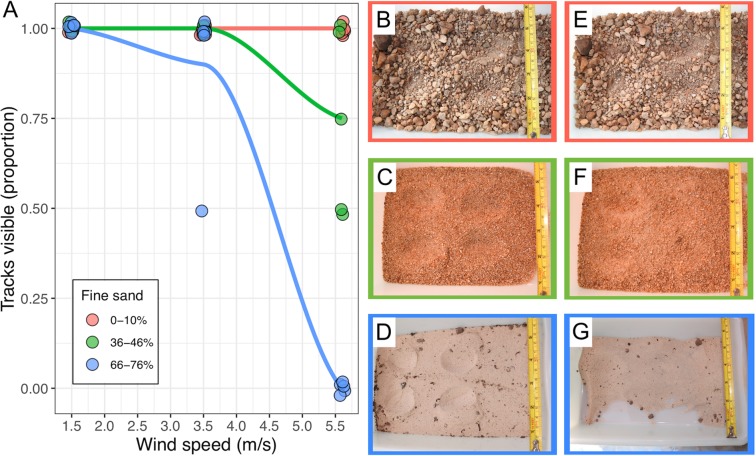
Track visibility in different substrates. (**A**) The proportion of experimental tracks visible in substrates with different amounts of fine sand was compared after 5 minutes exposure to three wind speeds (1.5, 3.5 and 5.6 m/s). Drawn tracks on different substrate types before (**B–D**), and after wind experiment (**E–G**), respectively. Photo credits: Fernanda Michalski.

In laboratory experiments, the conspicuousness (and therefore detectability) of tracks declined abruptly at a threshold of 66-76% fine sand (Fig. 3). The proportion of visible tracks declined with increasing wind speed such that at speeds of 5.6 m/s (equivalent to “moderate breeze” on the Beaufort scale) no tracks were visible in substrates with 66-76% fine sand (Fig. 3). Yet, the majority of tracks were still visible in substrates with lower proportions of fine sand, with 100% and 75% of tracks visible in substrates with 0-10% and 36-46% of fine sand, respectively (Fig. 3).

## Discussion

In this study across a large river extension of eastern Brazilian amazon we (1) quantified the grain size distribution of substrate types used by *Podocnemis unifilis* to lay nests and (2) showed that substrate grain size affects human removal of turtle nests. We first turn to explore the flexibility showed by *P. unifilis* in nesting areas in our study region and then demonstrate that nest-site substrate type affects nest removal rates by humans. Finally, we provide evidence to guide nest-site restoration by identifying which substrate type reduces nest removal by humans and will likely maximize turtle reproductive success along rivers accessible to humans.

Overall, *Podocnemis unifilis* laid nests in substrates with a wide variety of grain sizes, ranging from very fine sand to gravel. This diversity of grain sizes was expected, as compared with other freshwater turtles *P. unifilis* is considered a generalist species in terms of nest-site selection^17,34,40^. Thus, our results demonstrating that nests can be found in substrates ranging from over 76% of fine sand to over 93% of gravel across 88 km of waterways corroborates the generalist behavior for nest-site selection of this species.

We found that river segment (Araguari versus Falsino) and substrate grain size (represented by the first axis of the Principal Component Analysis) were the strongest predictors of nest removal by humans across all turtle nest-sites. Nests laid along the Araguari river were more likely to be removed by humans than nests along the Falsino river. This result is not surprising as the Araguari river has a larger number of houses and increased boat traffic compared with the Falsino^30,41,42^. Our findings highlight that even around protected areas with relatively low human disturbance, it is possible to have markedly different anthropogenic impacts and these differences should be considered in management plans and conservation initiatives in and around sustainable-use reserves.

Our study is the first to quantify how substrate type affects nest removal rates by humans. A study of marine turtle (*Caretta caretta* and *Chelonia mydas*) nesting in Syria identified that the visibility of tracks from these large (~160 kg) turtles depended on the texture of the beach substrate, with tracks hardly visible in dry coarse gravel^39^. The same study found that the longevity of a turtle track also depended on the substrate, with even a light breeze rapidly obliterating tracks on loose sand^39^. Our finding that turtle nests were less likely to be removed by humans in substrates with higher proportions of fine sand, also suggests that different substrates may produce differences in human detections of turtle tracks and nest marks. Our experimental manipulation of track longevity in different substrates exposed to different wind speeds corroborates this hypothesized link between human removal of turtle nests and track detectability. The reduced levels of nest removal that we found in nesting sites with more (>66%) fine sand could therefore be linked with the rapid obliteration of turtle tracks and nesting marks in the finer sand by even moderate breezes (i.e. of sufficient speed to raise dust or loose paper). Independent of the reason for reduced removal of fine-sand nests our results present compelling evidence that substrate grain size does affect turtle nest removal by humans and hence, must be considered an important variable in the management of turtle nesting areas with human activity.

Finally, due to the increasing number of new dams in the Amazon^19,22^ and the likely submersion of many freshwater turtle nesting areas^25^ baseline information for the construction of effective artificial nesting areas is required. Our results provide compelling evidence that substrate grain size does affect human removal of turtle nests and should be considered in the design of restored nesting areas. We suggest that using finer sand compared with coarse sand or gravel in restored nesting areas can increase nesting success along 164,971 km of rivers accessible to humans throughout the species range^28^. For example, as wind can obliterate tracks in loose sand^39^, combining the use of a thin layer of fine sand covering turtle nesting areas, could potentially reduce human removal. Also, as substrate texture of freshwater turtle nest-sites was related with nest temperature and sex ratio determination^38^, maintaining heterogeneous substrate types (i.e., mix of fine and coarse sand) in nesting areas is important.

Substrate type has also been shown to be an important factor for hatchling success in some turtle species^35,43^. We were unable to assess hatchling success but the results from previous studies enable us to generate informed expectations for likely outcomes. Differences in grain size did not influence the hatchling success of *P. unifilis* in previous studies^35^, but showed to be important for the larger congener *P. expansa*, with nests placed on fines sediments having improved hatching success^35^. Additionally, in loggerhead turtles (*Caretta caretta*), hatchlings from fine-grain sand nests had higher crawling and swimming performance, and fine-grain sand was recommended for use in hatchery incubation beds to increase hatchling survival and performance^43^. It therefore seems likely that the use of fine-grain sand substrates would not negatively affect *P. unifilis* hatchling success. It will be important for future studies to carefully monitor egg and hatchling survival as part of any conservation and restoration actions involving nesting area substrates.

Our results demonstrate that although *P. unifilis* uses a variety of substrate types, conservation initiatives must take nest substrate into consideration not only for gender determination, but also to reduce human removal of nests. Proportions of 66% or more of fine sand in nesting areas can directly reduce human detection of turtle nests. Therefore, our results can help to inform the successful restoration of *P. unifilis* nesting areas along the 164,971 km of rivers accessible to humans across the species range^28^.

## Methods

### Study area

**Figure 4:**
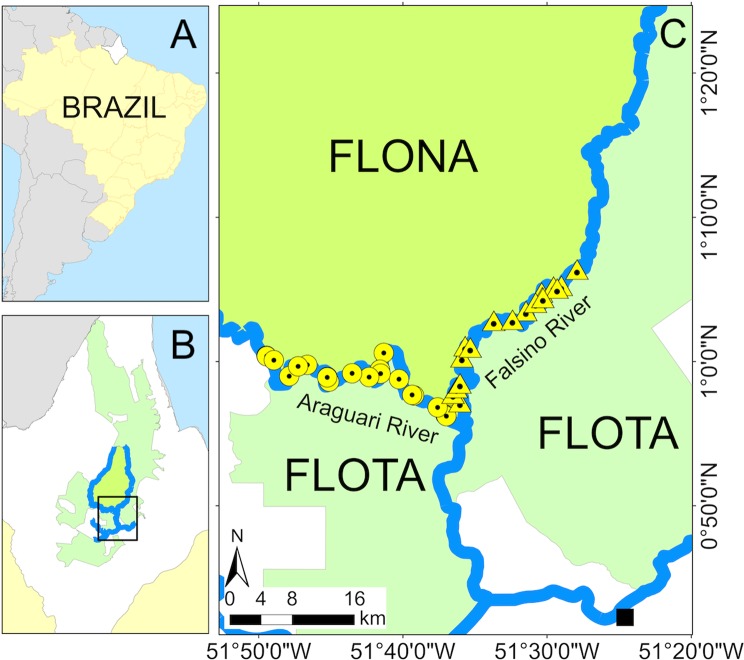
Study area. (**A**) State of Amapá in Brazil. (**B**) Location of the study area, FLONA and FLOTA within Amapá. (**C**) Location of the sampled nest sites along the Araguari (circles) and Falsino (triangles) rivers. Location of the nearest town (Porto Grande) is shown by a black square. Figure produced with ArcGIS 10.4 (https://desktop.arcgis.com/en/).

The study was conducted in the Araguari river basin, located in the Brazilian State of Amapá (Fig. 4). Climate in the area is characterized as equatorial monsoon^44^ with an annual rainfall greater than 2000 mm. The dry season extends between September and November (total monthly rainfall <150 mm), and the wet season from February to April (total monthly rainfall >300 mm)^45^.

This study was conducted along a total of 88 km of contiguous segments of Falsino and Araguari rivers, 42 km and 46 km, respectively (Fig. 4). These river segments were located between two sustainable-use protected areas, the National Forest of Amapá (hereafter FLONA), and the Amapá State Forest (hereafter FLOTA). Both segments can be classified as “clear-water”, with a low density of suspended particles^46^, but they experience markedly different human activity^41^. The Araguari river is characterized by higher human density (we counted 13 houses in 2017) and more intense boat and fishing activities (including commercial fishing)^41,42^. In contrast there is no commercial fishing and only 4 houses along the Falsino river.

### Turtle nest surveys and sampling

Monthly boat surveys were used to locate nesting areas. Nest searching was conducted between October and December 2017 during the nesting season of *Podocnemis unifilis* in the region^23,25^. While navigating along the rivers in a motorized boat at a constant speed (ca. 10 km/h) we performed an extensive search for nesting areas that involved identifying all suitable nesting areas through visually searching river banks, circling islands, stopping to search among boulders and rapids. Nesting areas were identified as areas>5 m^2^ of exposed substrate sufficiently raised above the river level not to be waterlogged at a depth of 15 cm, a representative depth that females dig when nesting^25,34,47^. Although we cannot guarantee that all nesting areas were located across 88 km of rivers, we assume that the comprehensive survey effort enables us to provide a robust and representative evaluation of the nest-site substrates.

In all nesting areas, a search for nests was done together with local residents with over 30 years of knowledge on nesting sites^25^. Searches were conducted by a team of three observers at a standardized speed (mean 0.8, range: 0.2-1.3 km per hour); time spent searching nesting areas ranged from 10 to 97 min depending on the size of the area. To minimize possible observer-related biases, at least one surveyor was constantly present with every search team throughout the entire study period. Logistical limitations hampered our capacity to visit all nesting areas more than once along 88 km of rivers. In order to standardize the number of visits for each nesting area, we included only data from a single visit during the first fortnight of November because this corresponded to the period when both turtle nesting and removal of nests by humans reached their peak during the nesting season^30^.

### Substrate collection and grain size determination

At each turtle nest we collected 200 g of substrate from the nest surface (1 – 3 cm), which was stored in plastic bags labeled with the identifications of the nesting area and the nest. The location of each turtle nest was obtained in the field with a handheld Global Positioning System (GPS) accurate to +2 m. We could not evaluate hatchling success from turtle nests surveyed due to high rates of nest harvest by humans in the study area. For example, in 2017, 42% of the nesting areas along 33 km of the Falsino River (the river segment with the lowest anthropogenic disturbance) were harvested by humans^23^. Thus, hatchling success in our study region could not be linked directly with nest substrate.

All substrate samples were analyzed at the Laboratório de Análises de Sedimentos do Instituto de Pesquisa Cientificas e Tecnológicas Estado do Amapá – IEPA (Macapá, Brazil). To obtain grain size frequency distribution of substrate samples we used dry sieving, which is a well-established technique^48^. In the laboratory, all organic material (i.e., vegetation) was removed manually from samples with tweezers. The samples were dried at 50 °C for 24 hours, and were shaken by a mechanical shaker for 10 minutes through a stack of 12 sieves^49^. The mesh size of the sieves were 2.0 mm, 1.4 mm, 1.0 mm, 0.71 mm, 0.5 mm, 0.355 mm, 0.25 mm, 0.18 mm, 0.125 mm, 0.09 mm, 0.063 mm, and <0.063 mm arranged in a decreasing geometric scale. After shaking, the amount of substrate retained on each sieve was weighed with a precision electronic scale with an accuracy of 0.001 g.

### Response variable

To quantify nest removal by humans we identified if each nest was removed or not by humans^30^, retaining individual nests as the sample unit in the analysis. Human removal of eggs was identified when an open nest was found with a typical depth between 10–15 cm, but lacking eggs. Signs of human activities in the nesting areas, such as footprints, charred wood from fires, campsite or trash were used for corroboration. Nests depredated by wild animals (identified by the presence of broken eggshells and/or partially eaten eggs around the nest, with animal excavation marks were present) though damaged were considered not removed by humans^30^.

### Explanatory variables

We examined a total of 8 (4 environmental, 2 anthropogenic and 2 spatial) variables that were likely to influence nest removal by humans (Table 4, Tables S1–S2). We measured four variables at each nesting area: (1) nesting area type (environmental: either island or bank); (2) river segment (anthropogenic: either Falsino or Araguari), (3) nest density (environmental: expressed as nests per m^2^); (4) distance to the nearest house (anthropogenic: km). We also recorded two variables at each individual nest: (1) distance to water (environmental: m), and (2) substrate grain sizes (environmental: PC1). We also included geographic coordinates of the nests and the identity of the nesting area to control for possible spatial autocorrelation in the models.

**Table 4:** Working hypotheses and variables used to explain *P. unifilis* nest removal by humans in the eastern Brazilian Amazon.

Category	Working hypothesis	Variable name	Variable description	Variable support^a^
Environmental	Nesting area type could influence the access of predators affecting nest removal	Type	Categorical – located along the river bank or island	−
Environmental	Nest density could influence the success of predators finding nests and affect nest removal	Nest density	Continuous – Number of turtle nests per nesting area (m^2^)	−
Environmental	Nest distance to water could influence the detectability of the nest, affecting nest removal	Distance to water	Continuous – Distance (m) from turtle nests to the nearest water source	+
Environmental	Different substrate types can affect nest detectability, and nest removal	PC1 scores	Continuous – Principal Component axis 1 scores from proportions of substrate grain sizes	++
Anthropogenic	Since Araguari river has higher anthropogenic pressure, different rivers will present differences in nest site selection and nest removal	River	Categorical – River segment (Falsino or Araguari)	++
Anthropogenic	Closer to houses human disturbances will increase, affecting nest site selection and nest removal	Distance to house	Continuous – Distance (km) to nearest riverine house	−
Spatial	Nests spatial distribution will affect nest removal	Long, Lat	Continuous – Coordinates (decimal degrees) of nests	−
Spatial	Nests located in the same nest site are more likely to have similar removal rates	Nesting site ID	Categorical – Nest site identification	++

^a^Strength of variable support from our information theoretic analysis. Unsupported = “−”, weakly supported “+”, and strongly supported = “++”.

Variables were selected based on previous studies conducted with *P. unifilis*, as well as our previous knowledge about the study area and species^23,25,30,42^. For example, nesting area type (island or bank) was found to be an important environmental variable for nest removal by humans at the scale of nesting areas in our study region^30^. River segment represents different intensities of human activity (i.e., high for Araguari and low for Falsino)^41^, and it was also highlighted as important predictor for nest removal by humans in previous studies^30^. Nest density was included as based on classical optimal foraging theory^50,51^ we would expect relatively increased effort and increased removal where nest density is greater and nests therefore more frequently/easily found. Distance to houses was an important variable to explain nest removal in our study area^30^, being more important than number of houses within radius of 1 or 5 km for explaining removal between nesting areas. Finally, nest distance to water and proportion of substrate grain size could both affect the detectability by humans. As all nesting sites were located along navigable rivers, and the main transport for riverine community is by boats and canoes, distance of nests from water edge as well as different substrate grain size may result in different detectability and consequent different rates of human removal.

Density of nests was obtained by dividing the total number of nests by the surface nesting site area. This was measured by mapping the surface nesting area using a GPS handheld *in situ*. Rocks and dense vegetation (non-nesting zones) were excluded from density calculations. Distance to the nearest house was obtained from the centroid point of each nest site to the nearest house (based on GPS fixes obtained *in situ* at the sampling period). Nest distance to the water’s edge was measured with a measuring tape at the day of the survey. Nesting area size was not included in our analysis as human removal of nests was not influenced by this variable in our study area^30^.

### Substrate type and track visibility

As humans often search for turtle nests by following the tracks of nesting females, previous studies suggest that differences in human removal of turtle nests can be explained by differences in detectability of the tracks in different substrates^39^. We experimentally tested different substrates to evaluate how the visibility of tracks was affected by different wind speeds. Following the dry sieving, we established three classes of substrate to represent the gradient of fine sand in the 216 nests: 0-10%, 36-46%, and 66-76%. We then selected five nests at random in each class.

In the laboratory samples were placed in tray providing a substrate depth of 6 mm. We drew four tracks in the tray, with size and depth following those recorded from tracks of adult females (Fig. S2). Based on the quantity of substrate collected and size of tray required to maintain a depth of 6 mm it was only possible to draw four tacks. Once drawn, tracks were exposed to different wind speeds (1.5, 3.5 and 5.6 m/s, measured using a digital anemometer) generated by a multispeed electric fan fixed at a constant height and distance from the tray. These speeds represent “light air”, “gentle breeze”, and “moderate breeze” on the Beaufort scale and are typical of those found in the study area during nesting season. The mean wind speed of the central point (0.924722, -51.595833) of our study area is 3.76 m/s according to the information obtained from the “*Global Wind Atlas 3.0, a free, web-based application developed, owned and operated by the Technical University of Denmark (DTU). The Global Wind Atlas 3.0 is released in partnership with the World Bank Group, utilizing data provided by Vortex, using funding provided by the Energy Sector Management Assistance Program (ESMAP). For additional information:* https://globalwindatlas.info”. The tracks were exposed to wind for five minutes, and after each exposure the number of visible tracks was noted and the tray then reset and the four tracks redrawn.

### Data analysis

All statistical analysis and graphics production were undertaken within the R language and environment for statistical computing^52^. The analysis of grain size samples followed the method of Folk and Ward^48^ with the packages “rysgran”^53^ and “soiltexture”^54^. Principal Component Analysis (PCA) of our seven substrate grain sizes (Table S3) was used to summarize the information of substrate grain size sampled on *P. unifilis* nests to avoid multicolinearity in subsequent analysis. The PCA was obtained from a scaled and centered data matrix of our seven substrate grain sizes, and axes derived from the squared correlations coefficients with the variables. Based on the Kaiser-Guttman criterion only the first three principal components (with eigenvalues >1) should be interpreted^55^ (Table S3). Component loadings, summary of principal components after varimax rotation (Table S4), and inspection of correlations revealed that the three axes with eigenvalues >1 (PC1, PC2, and PC3, representing 90% of the variance) were generally represented by proportion of fine sand (negative correlation), medium sand (negative correlation), and gravel (positive correlation), respectively (Fig. S1).

We used Generalized Additive Models (GAMs) to assess the effects of environmental and anthropogenic variables on turtle nests removed by humans. This approach allows the shape of the relationship between the response and the explanatory variables to be determined from the data, rather than following a prescribed functional form^56^. Thus, we analyzed the response of the nests removed by humans (binary variable), and examined the influence of the explanatory variables (nesting area type, river segment, nest density, distance to the nearest house, distance to water, and scores of PCA first axis related to substrate grain sizes) during the peaks of turtle nesting season and nest removal. A two-step process was adopted with GAMs run (with default settings) using the R package “mgcv”^57^. Firstly we established whether variables should be include as non-parametric (non-linear) smoothed terms by checking estimated degrees of freedom (EDF) values (typically an EDF value close to or less than 1 suggests linear relationship) and standard diagnostic plots^56^. Based on results from this preliminary model, we then re-fitted the model including variables as parametric (i.e. linear) terms as appropriate. To control for spatial autocorrelation within and between nesting areas we included the geographic coordinates of the nests as a non-parametric term and the nesting area ID as a random effect (penalized smoothed regression term)^56^.

## Supplementary information

Supplementary information.

## Data Availability

The raw data was supplied as a Supplementary Information File.

## References

[1] Gibbons, J. W. *et al*. The Global Decline of Reptiles, Déjà Vu Amphibians: Reptile species are declining on a global scale. Six significant threats to reptile populations are habitat loss and degradation, introduced invasive species, environmental pollution, disease, unsustainable use, and global climate change. *BioScience* **50**, 653–666, 10.1641/0006-3568(2000)050%5B0653:TGDORD%5D2.0.CO;2 (2000).

[2] Lovich JE, Ennen JR, Agha M, Gibbon JW (2018). Where Have All the Turtles Gone, and Why Does It Matter?. BioScience.

[3] Eisemberg CC, Rose M, Yaru B, Georges A (2011). Demonstrating decline of an iconic species under sustained indigenous harvest - The pig-nosed turtle (*Carettochelys insculpta*) in Papua New Guinea. Biological Conservation.

[4] Harju E, Sirén AH, Salo M (2018). Experiences from harvest-driven conservation: management of Amazonian river turtles as a common-pool resource. Ambio.

[5] Rhodin AGJ (2018). Global Conservation Status of Turtles and Tortoises (Order Testudines). Chelonian Conservation and Biology.

[6] Young TP (2000). Restoration ecology and conservation biology. Biological Conservation.

[7] Smallwood KS (2001). Linking Habitat Restoration to Meaningful Units of Animal Demography. Restoration Ecology.

[8] Palis JG (2007). If you build it, they will come: herpetofaunal colonization of constructed wetlands and adjacent terrestrial habitat in the Cache River drainage of southern Illinois. Transactions of the Illinois State Academy of Science.

[9] Brock KA, Reece JS, Ehrhart LM (2009). The Effects of Artificial Beach Nourishment on Marine Turtles: Differences between Loggerhead and Green Turtles. Restoration Ecology.

[10] Grain DA, Bolten AB, Bjorndal KA (1995). Effects of Beach Nourishment on Sea Turtles: Review and Research Initiatives. Restoration Ecology.

[11] Dowling Z, Hartwig T, Kiviat E, Keesing F (2010). Experimental Management of Nesting Habitat for the Blanding’s Turtle (*Emydoidea blandingii*). Ecological Restoration.

[12] Roosenburg WM (2014). Nesting Habitat Creation Enhances Recruitment in a Predator-Free Environment: Malaclemys Nesting at the Paul S. Sarbanes Ecosystem Restoration Project. Restoration Ecology.

[13] Páez VP, Lipman A, Bock BC, Heppell SS (2015). A Plea to Redirect and Evaluate Conservation Programs for South America’s Podocnemidid River Turtles. Chelonian Conservation and Biology.

[14] Brooks RJ, Brown GP, Galbraith DA (1991). Effects of a sudden increase in natural mortality of adults on a population of the common snapping turtle (*Chelydra serpentina*). Canadian Journal of Zoology.

[15] Gibbs, J. P. & Amato, G. D. In Turtle Conservation (ed. Klemens, M. W.) Ch. 8, 207-217 (Smithsonian Institution Press, 2000).

[16] Spencer, R. J. Experimentally testing nest site selection: Fitness trade-offs and predation risk in turtles. *Ecology* **83**, 2136–2144, 10.1890/0012-9658(2002)083%5B2136:ETNSSF%5D2.0.CO;2 (2002).

[17] Moll, D. & Moll, E. O. The ecology, exploitation and conservation of river turtles. (Oxford University Press, 2004).

[18] Refsnider JM, Janzen FJ (2010). Putting Eggs in One Basket: Ecological and Evolutionary Hypotheses for Variation in Oviposition-Site Choice. Annual Review of Ecology, Evolution, and Systematics.

[19] Castello L (2013). The vulnerability of Amazon freshwater ecosystems. Conservation Letters.

[20] Smith, N. J. H. Aquatic turtles of Amazonia: an endangered resource. *Biological Conservation*, 165–176, 10.1016/0006-3207(79)90019-3 (1979).

[21] Fagundes CK, Vogt RC, de Souza RA, De Marco P (2018). Vulnerability of turtles to deforestation in the Brazilian Amazon: Indicating priority areas for conservation. Biological Conservation.

[22] Lees AC, Peres CA, Fearnside PM, Schneider M, Zuanon JAS (2016). Hydropower and the future of Amazonian biodiversity. Biodiversity and Conservation.

[23] Norris D, Michalski F, Gibbs JP (2018). Community involvement works where enforcement fails: conservation success through community-based management of Amazon river turtle nests. PeerJ.

[24] Fearnside PM (2014). Impacts of Brazil’s Madeira River Dams: Unlearned lessons for hydroelectric development in Amazonia. Environmental Science & Policy.

[25] Norris D, Michalski F, Gibbs JP (2018). Beyond harm’s reach? Submersion of river turtle nesting areas and implications for restoration actions after Amazon hydropower development. PeerJ.

[26] IUCN. IUCN redlist 2019-2 update, https://www.iucn.org/news/species/201907/unsustainable-fishing-and-hunting-bushmeat-driving-iconic-species-extinction-iucn-red-list (2019).

[27] Tortoise & Freshwater Turtle Specialist Group. Podocnemis unifilis (errata version published in 2016). The IUCN Red List of Threatened Species 1996: e.T17825A97397562. http://dx.doi.org/10.2305/IUCN.UK.1996.RLTS.T17825A7506933.en. Accessed on 31/01/2019. 1996).

[28] Norris D, Peres CA, Michalski F, Gibbs JP (2019). Prospects for freshwater turtle population recovery are catalyzed by pan-Amazonian community-based management. Biological Conservation.

[29] Peres CA (2000). Effects of Subsistence Hunting on Vertebrate Community Structure in Amazonian Forests. Conservation Biology.

[30] Quintana I (2019). Nest removal by humans creates an evolutionary trap for Amazonian freshwater turtles. Journal of Zoology.

[31] Alho CJR (1985). Conservation and management strategies for commonly exploited amazonian turtles. Biological Conservation.

[32] Magnusson, W. E. *et al*. Effects of Brazil’s Political Crisis on the Science Needed for Biodiversity Conservation. *Frontiers in Ecology and Evolution* **6**, 10.3389/fevo.2018.00163 (2018).

[33] Escobar H (2019). Brazilian scientists lament ‘freeze’ on research budget. Science.

[34] Escalona T, Valenzuela N, Adams DC (2009). Nesting ecology in the freshwater turtle *Podocnemis unifilis*: spatiotemporal patterns and inferred explanations. Functional Ecology.

[35] Ferreira PD, Castro PTA (2010). Nesting ecology of Podocnemis expansa (Schweigger, 1812) and Podocnemis unifilis (Troschel, 1848) (Testudines, Podocnemididae) in the Javaes River, Brazil. Brazilian Journal of Biology.

[36] Ferreira Júnior, P. D. & Castro, P. T. A. Thermal environment characteristics of *Podocnemis expansa* and *Podocnemis unifilis* nesting areas on the Javaés River, Tocantins, Brazil. *Chelonian Conservation and Biology* **5**, 102–107, 10.2744/1071-8443(2006)5%5B102:TECOPE%5D2.0.CO;2 (2006).

[37] Páez VP, Bock BC (1998). Temperature effect on incubation period in the Yellow-Spotted River Turtle, *Podocnemis unifilis*, in the Colombian Amazon. Chelonian Conservation and Biology.

[38] de Souza RR, Vogt RC (1994). Incubation temperature influences sex and hatchling size in the Neotropical turtle *Podocnemis unifilis*. Journal of Herpetology.

[39] Kasparek M (1995). The nesting of marine turtles on the coast of Syria. Zoology in the Middle East.

[40] Ferreira Júnior PD, Castro PdTA (2003). Geological control of *Podocnemis expansa* and *Podocnemis unifilis* nesting areas in Rio Javaés, Bananal Island, Brazil. Acta Amazonica.

[41] de Oliveira IAP, Norris D, Michalski F (2015). Anthropogenic and seasonal determinants of giant otter sightings along waterways in the northern Brazilian Amazon. Mammalian Biology - Zeitschrift für Säugetierkunde.

[42] Norris D, Michalski F (2013). Socio-economic and spatial determinants of anthropogenic predation on Yellow-spotted River Turtle, *Podocnemis unifilis* (Testudines: Pelomedusidae), nests in the Brazilian Amazon: Implications for sustainable conservation and management. Zoologia (Curitiba).

[43] Saito T, Wada M, Fujimoto R, Kobayashi S, Kumazawa Y (2019). Effects of sand type on hatch, emergence, and locomotor performance in loggerhead turtle hatchlings. J. Exp. Mar. Biol. Ecol..

[44] Kottek M, Grieser J, Beck C, Rudolf B, Rubel F (2006). World map of the Koppen-Geiger climate classification updated. Meteorologische Zeitschrift.

[45] Paredes OSL, Norris D, de Oliveira TG, Michalski F (2017). Water availability not fruitfall modulates the dry season distribution of frugivorous terrestrial vertebrates in a lowland Amazon forest. Plos One.

[46] Junk WJ, Wittmann F, Schöngart J, Piedade MT (2015). A classification of the major habitats of Amazonian black-water river floodplains and a comparison with their white-water counterparts. Wetlands Ecology and Management.

[47] Pignati MT, Fernandes LF, Miorando PS, Ferreira PD, Pezzuti JCB (2013). Nesting site and hatching success of *Podocnemis unifilis* (Testudines: Podocnemididae) in a floodplain area in lower Amazon River, Pará, Brazil. *South American*. Journal of Herpetology.

[48] Folk RL, Ward WC (1957). Brazos river bar: A study in the significance of grain size parameters. Journal of Sedimentary Research.

[49] Carter, M. R. & Gregorich, E. G. Soil sampling and methods of analysis. 2nd edn, (CRC Press, 2008).

[50] Stephens, D. W. & Krebs, J. R. Foraging Theory. 247 (Princeton University Press, 1986).

[51] Wilke, A., Hutchinson, J. M. C. & Todd, P. M. In Proceedings of the Annual Meeting of the Cognitive Science Society. (2004).

[52] R: A language and environment for statistical computing (Version 3.6.0), https://www.R-project.org/ v. 3.6.0 (R Foundation for Statistical Computing, Vienna, Austria, 2019).

[53] rysgran: Grain size analysis, textural classifications and distribution of unconsolidated sediments, https://CRAN.R-project.org/package=rysgran (2014).

[54] soitexture: Functions for soil texture plot, classification and transformation, https://CRAN.R-project.org/package=soiltexture (2018).

[55] Legendre, P. & Legendre, L. Numerical Ecology. 3rd edn, (Elsevier, 2012).

[56] Wood, S. N. Generalized Additive Models: An Introduction with R. 2nd edn, (Chapman and Hall/CRC, 2017).

[57] mgcv: Mixed Gam Computation Vehicle with Automatic Smoothness Estimation (Version 1.8-28), https://cran.r-project.org/web/packages/mgcv/index.html (2019).

