# Supplementary material for: Substrate influences human removal of freshwater turtle nests in the eastern Brazilian Amazon
Source: Sci Rep. 2020 May 15;10:8082. doi: 10.1038/s41598-020-65074-1 (PMC7228982; doi:10.1038/s41598-020-65074-1)
Supplement: Supplementary file 1 — Supplementary information. [file 41598_2020_65074_MOESM1_ESM.pdf]

Supplementary Information

Substrate influences human removal of freshwater turtle nests in the eastern Brazilian Amazon

Fernanda Michalski[1,2,3,*], Darren Norris[1,2,4], Itxaso Quintana[5], Andressa Valerio[1,2] & James P. Gibbs[6]

[1] Postgraduate Programme in Tropical Biodiversity, Federal University of Amapá, Macapá, Amapá, Brazil
[2] Ecology and Conservation of Amazonian Vertebrates Research Group, Federal University of Amapá, Macapá, Amapá, Brazil
[3] Pro-Carnivores Institute, Atibaia, São Paulo, Brazil
[4] School of Environmental Sciences, Federal University of Amapá, Macapá, Amapá, Brazil
[5] Ecology Department, Federal University of Rio Grande do Sul, Porto Alegre, Rio Grande do Sul, Brazil
[6] Department of Forest and Environmental Biology, State University of New York, College of Environmental Science and Forestry, Syracuse, NY, USA
* Corresponding Author: fmichalski@gmail.com

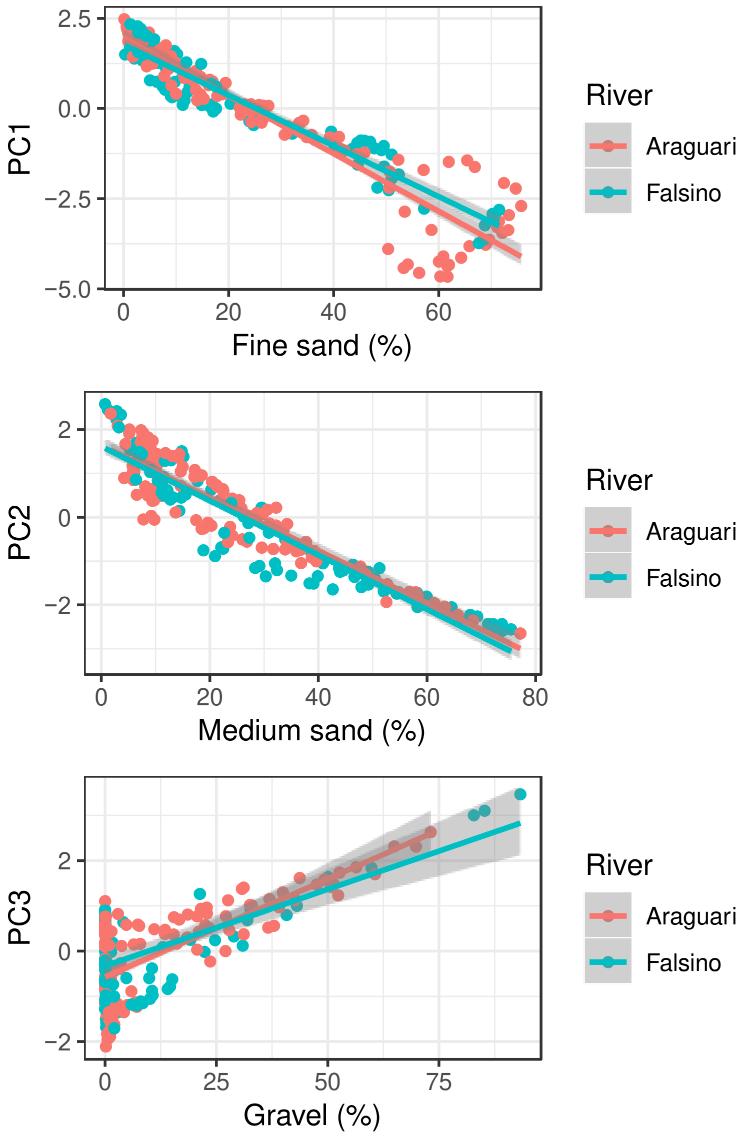

Supplementary Figure S1. Relationship between proportion of grain size substrates and principal components. Different colors represent substrates sampled in Araguari (red) and Falsino (green) rivers.

Supplementary Table S1. *Podocnemis unifilis* nests sampled in the study area with coordinates and proportion of each grain size particle.

Nest_ID	Long	Lat	Gravel	Very coarse sand	Coarse sand	Medium sand	Fine sand	Very fine sand	Coarse silt
2012P041N1	-51.52449698	1.058680974	0	0.218939488	11.42303182	75.49070493	11.58507798	1.171154086	0.111091703
2012P041N2	-51.52442104	1.058639986	0.013322137	0.321078464	12.88919225	73.81082466	11.26638635	1.498266376	0.200929755
2012P041N3	-51.52431903	1.058321977	0.053120969	1.599863931	51.54030859	42.63025112	3.537457516	0.611863783	0.027134091
2012P041N4	-51.52449396	1.058707964	0	0.274825322	22.41230683	72.00687837	5.036651691	0.253274307	0.016063488
2012P041N5	-51.52446304	1.058662031	0	0.283468323	17.39565412	71.24969562	9.917050213	1.025395888	0.128735834
2012P041N6	-51.52450101	1.058652978	0.003903552	0.482113333	16.78334498	72.18996165	9.223400754	1.1633078	0.153967936
2012P041N7	-51.52429397	1.058299011	0	0.778251371	33.26448619	52.01841746	9.004635458	4.757589072	0.176620448
2012P041N8	-51.5244	1.05849498	0.058531664	0.535824425	24.58949177	66.11061586	7.406053433	1.225469265	0.074013589
2012P041N9	-51.52429297	1.058367994	0	0.792668157	33.56256165	58.25154516	6.359824695	0.898999863	0.134400479
2012P05N1	-51.50620398	1.080049966	0.109597839	0.478929591	11.57423982	59.90120239	23.92199705	3.815779895	0.198253415
2012P05N2	-51.50621597	1.080054995	0.038103264	0.354136221	12.70502296	57.23722943	24.75457015	4.686751321	0.224186657
2012P06N1	-51.49288103	1.086413004	0.002744774	0.698220588	7.381196202	64.52878719	24.6242654	2.618713969	0.146071879
2012P06N2	-51.49286301	1.086403029	0.048812357	0.942657459	9.960865266	69.27421814	17.61452312	2.02790889	0.131014763
2012P06N3	-51.49284901	1.086419038	0.153658801	1.556543695	11.53284133	67.97940174	16.78428051	1.893495493	0.099778442
2012P06N4	-51.49285999	1.086403029	0.058553708	0.653874703	4.788724942	58.11682688	32.11299019	4.051637083	0.217392498
2012P07N1	-51.49259102	1.086914996	24.68593914	37.06803384	12.91164303	14.73713347	9.105857299	1.368407141	0.122986078
2012P07N10	-51.49243302	1.086867973	50.11576764	19.5472742	6.380587746	14.82978017	7.964225255	1.0432555	0.119109492
2012P07N11	-51.49239496	1.086796978	93.26367426	4.753281509	0.88309854	0.698868235	0.312253909	0.078001136	0.010822408
2012P07N2	-51.49260602	1.086909967	1.657714456	8.788721513	17.47038584	49.16031228	20.35783554	2.355657461	0.209372919
2012P07N3	-51.49258599	1.086929999	28.91901704	32.96801553	19.66911453	12.84794289	5.043819186	0.523815555	0.028275268
2012P07N4	-51.49259596	1.086928993	12.97601799	26.25448029	16.71855417	29.52309393	12.73608816	1.645981512	0.14578394
2012P07N5	-51.49259202	1.08693704	31.94931496	33.07163088	10.93762938	15.14530397	7.86369391	0.973764575	0.05866232

Nest_ID	Long	Lat	Gravel	Very coarse sand	Coarse sand	Medium sand	Fine sand	Very fine sand	Coarse silt
2012P07N6	-51.49253301	1.086912984	22.33833998	26.25479505	25.53786908	17.74621259	6.771388807	1.17770007	0.173694424
2012P07N7	-51.49239496	1.086893035	59.82778527	16.68891876	9.848857014	7.777442514	4.899316976	0.856784477	0.100894988
2012P07N8	-51.49242103	1.086898986	85.24236063	7.728562478	1.737689413	2.849323761	2.040789857	0.348645327	0.052628533
2012P07N9	-51.49240402	1.086911978	82.81919014	8.324077542	2.569111708	3.620506411	2.274056869	0.353307721	0.039749612
2012P081N1	-51.59826402	1.005062023	1.359538629	0.865120114	3.588489286	33.33150345	48.3042448	11.95966799	0.591435735
2012P081N2	-51.59824097	1.005053977	0.097016106	0.591578663	3.870812874	33.88377331	50.99918105	10.08593212	0.471705882
2012P081N3	-51.598247	1.005046014	0.366831048	3.294282776	9.76596467	38.73018189	40.19710903	7.36565576	0.279974827
2012P081N4	-51.59825404	1.005054982	0.158714468	0.923529482	3.397208543	32.55014558	50.48587896	11.88091872	0.603604251
2012P081N5	-51.59826502	1.005074009	0.115794904	0.466921372	2.337251036	36.14781636	52.37596575	8.114922825	0.441327755
2012P091N1	-51.59472601	1.017917013	0.00925022	0.12404152	1.323027538	46.38641123	44.5759266	7.178367867	0.402975028
2012P12N1	-51.505661	1.074034022	0.033475834	2.156123109	56.41810569	38.35756881	2.597694807	0.405800948	0.031230808
2012P12N2	-51.50567802	1.074010972	0.00603731	0.867476564	67.46174541	30.31358085	1.222230871	0.119997765	0.008931227
2012P13N1	-51.48345399	1.087784031	4.108530961	1.198415086	15.23386259	59.92978559	16.35987779	3.020087848	0.149440136
2012P13N2	-51.48338098	1.087816972	21.28796632	2.542731665	14.45139857	51.24588416	9.733898551	0.708550023	0.029570706
2012P13N3	-51.48313103	1.087771961	0.337990007	7.198099754	26.06024662	54.41853598	11.57171838	0.393207678	0.020201587
2012P13N4	-51.48341501	1.087549003	0.322543457	0.661056883	31.38523007	49.20831377	14.89234495	3.232670962	0.297839912
2012P13N5	-51.48340403	1.087554032	0.224492882	0.972686034	36.28624489	47.91989727	12.15880002	2.227111134	0.210767773
2012P16N1	-51.48826897	1.08461299	0.026193474	0.033477755	0.936878217	39.67827428	49.60984196	9.459486428	0.25584788
2012P16N2	-51.48825497	1.084601004	0.024106283	0.051306955	0.776990927	50.42205959	43.53410091	4.963648424	0.227786908
2012P16N3	-51.48821197	1.084648026	0	0.041532468	0.297316563	26.24083241	57.19954354	15.60357858	0.617196439
2012P17N1	-51.50551197	1.074430989	0.009681764	0.105052134	17.0553762	73.9571018	7.910700196	0.911882469	0.050205438
2012P241N1	-51.56149403	1.047446961	32.68236405	9.916686045	14.99880738	20.24580012	18.14563575	3.856663669	0.154042992
2012P242N1	-51.56146998	1.047304971	0.177770194	7.074485362	63.33732979	22.45088369	5.471310545	1.330257904	0.157962513
2012P242N2	-51.56143402	1.047315029	3.025792785	28.37645266	45.72068017	12.71024504	8.048779393	2.037645465	0.080404496
2012P242N3	-51.56142999	1.047319975	1.516676003	23.85821544	51.22218525	14.38228277	7.168843193	1.788294897	0.063502452
2012P242N4	-51.56147903	1.047328021	1.025569632	3.359135993	29.14368204	43.80544066	17.07980362	5.275348955	0.311019098

Nest_ID	Long	Lat	Gravel	Very coarse sand	Coarse sand	Medium sand	Fine sand	Very fine sand	Coarse silt
2012P28N1	-51.46536701	1.106505012	15.07353106	45.48931185	20.27210009	6.419772666	10.19475121	2.395725638	0.154807486
2012P28N2	-51.46537196	1.106503	30.91812642	47.62745272	14.10964889	2.807954841	3.692834678	0.765978161	0.078004287
2012P28N3	-51.46540096	1.106481962	6.601667141	47.07277652	28.18465253	5.318387932	9.667020847	3.009932007	0.145563022
2012P28N4	-51.465394	1.106522027	14.07623332	66.07998029	14.09673392	1.210782619	3.255954777	1.179258576	0.101056504
2012P28N5	-51.46517498	1.106601991	40.7639141	27.02955986	15.64591006	7.723080514	6.951316099	1.78023517	0.105984192
2012P28N6	-51.46544999	1.106512975	1.964709294	13.37277923	11.84944083	14.7449017	47.71826125	10.07222932	0.277678381
2017A1N1	-51.65352398	0.960939014	0.335498181	0.899980851	5.877750606	9.35216139	56.26855014	25.86582267	1.400236165
2017A1N2	-51.65351903	0.960892998	6.79228362	5.118416183	6.135734942	9.037507519	50.36825183	21.17136305	1.376442856
2017A1N3	-51.65353102	0.960914958	3.561906495	2.62322765	5.511548454	10.28292022	54.10665641	22.43801319	1.475727583
2017A1N4	-51.65355801	0.960914958	0.178723438	0.913219117	5.060655677	9.257974374	64.26278211	19.01321601	1.313429274
2017A1N5	-51.65354401	0.960939014	0.053720162	5.143881311	8.662308463	14.61648241	53.51848125	17.17324833	0.831878074
2017A1N6	-51.65353403	0.960931974	4.213644355	3.451595095	5.587372647	9.084390998	53.32299993	22.78691706	1.55307991
2017A2N1	-51.688676	1.010137014	0.19421527	0.363074242	0.490155217	10.85903445	60.2832019	26.50601688	1.304302035
2017A3N1	-51.69307499	0.990793006	0	0.105162553	7.917102347	77.1962545	14.02791551	0.703330747	0.050234344
2017A3N2	-51.69305999	0.990837011	0	0.379429883	11.72337083	63.29750355	22.29837644	2.124547971	0.17677133
2017A3N3	-51.693033	0.99084598	0	0.105162553	7.917102347	77.1962545	14.02791551	0.703330747	0.050234344
2017A4N1	-51.75388201	0.982297026	0	0	0.259119772	17.46403321	72.39787449	9.66815984	0.210812693
2017A4N2	-51.75387598	0.982315969	0	0.053522189	0.217779941	25.98260354	66.88433022	6.727883813	0.133880294
2017A4N3	-51.75389098	0.982294008	0	0.014852599	0.052709224	13.68894557	74.66241592	11.38429225	0.196784437
2017A4N4	-51.75383298	0.982293002	0	0.084198443	0.302904771	31.68292074	61.90437213	5.872080256	0.153523657
2017A5N1	-51.79786496	0.983163966	0	0.487412906	0.763590276	8.554315963	75.70187808	14.10484764	0.387955135
2017A5N2	-51.79787099	0.983206965	0.011514139	0.127253668	0.910514204	8.524799512	73.35817596	16.59989961	0.467842903
2017A6N1	-51.61730904	0.938197989	0.001012742	0.78436893	2.177446634	7.109147291	61.94671355	26.90000587	1.081304979
2017A6N2	-51.61732698	0.938180974	0	0.617284258	1.709985604	10.21079617	60.02063432	26.37687201	1.064427642
2017A6N3	-51.61733301	0.938188015	0.382124304	0.886103414	2.115974053	9.108634333	60.87717504	25.62367664	1.006312209
2017A6N4	-51.61733603	0.93818902	0.166500316	0.693485289	1.397545193	7.680263527	61.62272465	27.35957334	1.079907682

Nest_ID	Long	Lat	Gravel	Very coarse sand	Coarse sand	Medium sand	Fine sand	Very fine sand	Coarse silt
2017A6N5	-51.61728901	0.93819296	0.101869012	0.566066444	1.1564278	6.898507634	61.6724577	28.39002562	1.214645791
2017P2N1	-51.60809197	0.961305974	43.08632064	19.45631027	20.55619396	11.71401328	4.48890062	0.665927856	0.032333377
2017P3N1	-51.58867302	1.016425034	0.055939659	0.14406583	1.292300936	49.04984917	45.14764627	4.214936039	0.095262096
2017P3N2	-51.58867302	1.016388992	0.018206804	0.112632779	0.899466017	49.00628259	45.83824875	4.031385538	0.093777513
2017P3N3	-51.58869398	1.016392009	0.042455504	0.132411291	1.548747308	48.99903639	44.48276839	4.661206212	0.133374901
2017P3N4	-51.588727	1.016460992	0	0.052465609	2.233877922	54.78337239	39.53258728	3.330867982	0.066828818
2017P3N5	-51.58873203	1.016397038	0	0.036012346	1.141421779	43.97531409	49.52395868	5.240145486	0.083147619
2017P3N6	-51.58870202	1.016328977	0	0.120734549	2.027432048	46.64081946	46.92810679	4.18073947	0.102167682
2017P4N1	-51.53996998	1.048580026	0.018659248	0.048693653	0.678917245	44.54875552	49.09402864	5.463268222	0.147677472
2017P4N2	-51.53996402	1.048525041	0.023656696	0.035335319	0.676162285	40.84757851	51.03587882	7.262955161	0.118433208
2017P5N1	-51.51324302	1.068134001	4.7754661	15.04410822	32.9556622	30.85638565	12.54120718	3.571230713	0.255939948
2017P5N2	-51.51325098	1.068112962	10.54660283	15.26866132	33.0524531	27.07787077	11.16912759	2.69551717	0.189767218
2017P5N3	-51.51332701	1.067958986	0.632317578	9.116805422	55.14051696	32.01366488	2.804453407	0.265905578	0.026336174
2017P5N4	-51.51333396	1.067974996	0.440317236	6.19980835	47.26379277	39.87627021	5.434902436	0.725697876	0.05921112
2017P6N1	-51.50478903	1.073815003	0.01750888	3.640999114	64.55334647	28.35959749	2.967381105	0.443059475	0.018107475
2017P6N2	-51.50477101	1.073846016	0.151502529	4.98132533	60.66267017	29.13153682	4.448996227	0.603416065	0.020552862
2017P6N3	-51.50477998	1.07379497	0.040843317	1.542471066	52.01433855	39.10276358	6.495982494	0.774676545	0.028924449
2017P6N4	-51.504783	1.073747026	0.261715862	5.296466661	71.11598597	20.95841775	2.169393559	0.188493281	0.009526916
2017P6N5	-51.50477101	1.073872	0.295289688	5.910834192	64.89096939	22.09497479	4.796049496	1.778425438	0.233457015
2017P6N6	-51.50477202	1.073875017	0.219782412	6.90122758	72.0552174	18.81964826	1.630987974	0.350095877	0.023040498
A181N1	-51.65579002	0.961590959	73.12642379	8.696375265	4.536856074	7.322914825	5.501061851	0.760400043	0.055968152
A181N10	-51.65604902	0.961506972	11.04198206	8.699084197	9.056444104	33.49518148	33.41479795	4.044873068	0.247637143
A181N11	-51.65607601	0.96152097	23.35281606	18.57378253	17.33997633	24.1207939	14.65467453	1.842897241	0.115059404
A181N12	-51.65614801	0.961491968	32.91169965	12.55785231	13.39980722	22.49673794	16.23697544	2.257124943	0.139802492
A181N13	-51.656059	0.961479982	18.41949598	15.95699064	14.18230556	24.20903666	23.03646261	3.908080977	0.287627572
A181N2	-51.65576597	0.961556006	52.64869886	13.02960412	10.07078563	14.20227894	8.531911282	1.433584519	0.083136651

Nest_ID	Long	Lat	Gravel	Very coarse sand	Coarse sand	Medium sand	Fine sand	Very fine sand	Coarse silt
A181N3	-51.65600603	0.961490963	0.888961031	4.456741875	7.486870856	34.23696138	45.84055239	6.755584412	0.33432806
A181N4	-51.65601198	0.961491968	7.900881668	14.27598438	13.83970438	33.595862	26.3453468	3.827433695	0.21478708
A181N5	-51.65602597	0.961514013	13.45456015	12.23362722	12.05066939	32.17463367	26.48248479	3.435436143	0.16858863
A181N6	-51.65601902	0.961528011	22.48444127	22.45167291	10.72389608	22.40671632	19.41070896	2.413070181	0.109494283
A181N7	-51.65607601	0.961490963	15.3873495	14.74801495	12.95015135	28.68550822	24.25732505	3.747173468	0.224477466
A181N8	-51.65603897	0.961496998	21.07991272	19.42917134	16.21877082	25.40990754	15.59499685	2.112032176	0.155208555
A181N9	-51.65607098	0.961507978	13.44404298	12.26945281	13.9443962	31.00765078	25.77828973	3.361785673	0.194381825
A19N1	-51.67105297	0.979786981	39.08627569	24.40005645	11.59645696	11.82010345	11.09206805	1.908699375	0.096340026
A19N2	-51.671045	0.979797961	50.79051137	26.40541038	8.980971731	7.70523376	5.190340085	0.873283116	0.054249557
A19N3	-51.67107501	0.979783041	51.30329225	21.8504726	9.153705033	8.181732073	7.733309404	1.718662758	0.058825875
A19N4	-51.671116	0.97974197	42.66114697	15.64643237	8.090956242	14.37305098	15.2623563	3.583528558	0.382528582
A37N1	-51.69233898	0.986185977	0	0.064734603	7.32548339	57.87692446	30.66694595	3.777697982	0.288213614
A37N2	-51.692415	0.986181032	0.028355844	0.354572642	11.68051485	58.2900479	26.26837586	3.159758023	0.218374886
A37N3	-51.69238198	0.986173991	0	0.128449513	7.712832177	52.70738409	34.84418483	4.358015445	0.249133943
A37N4	-51.69243503	0.986123029	0.078848404	0.238043281	38.68670218	52.5226246	7.742923243	0.657253129	0.07360516
A40C2N1	-51.70550301	0.982257966	0.932844658	31.17336502	50.08947032	8.097650039	7.105424409	2.37633576	0.224909794
A40C2N2	-51.70552899	0.982277999	1.020789673	35.60037009	48.03673587	5.958486458	6.996673971	2.193723218	0.193220724
A49C2N1	-51.72531296	0.986617981	2.07225043	32.40255649	52.8574001	10.92824788	1.477870107	0.209349968	0.052325022
A49C2N10	-51.72528799	0.986638013	2.467301218	21.34036793	53.65356518	20.27649033	2.005818221	0.214712929	0.041744188
A49C2N11	-51.72523803	0.986655029	1.915236153	14.36167611	50.52165208	26.84389762	5.468388972	0.745224386	0.143924672
A49C2N12	-51.72514298	0.986694004	7.673639425	5.572491233	13.73175483	47.75291036	22.1204974	2.982130147	0.166576616
A49C2N13	-51.72512999	0.986681012	3.454085174	9.313210078	32.65759011	39.61057269	13.3077368	1.522168832	0.134636315
A49C2N14	-51.72510501	0.986731974	3.270330236	8.414438408	33.24399177	38.75451344	13.76565203	2.30530167	0.245772449
A49C2N15	-51.72519897	0.986710014	0.007377017	0.195690327	19.80684178	68.47755817	9.980057132	1.430592988	0.101882585
A49C2N16	-51.72519101	0.986718982	0	0.984798226	25.68971568	63.47081472	9.095855591	0.678146994	0.080668792
A49C2N17	-51.72511297	0.986738009	0.448194449	13.56922042	45.58022296	36.79360603	3.44131312	0.156216945	0.011226066

Nest_ID	Long	Lat	Gravel	Very coarse sand	Coarse sand	Medium sand	Fine sand	Very fine sand	Coarse silt
A49C2N2	-51.72534599	0.986654023	0.643364974	29.93937023	59.27897824	9.376685303	0.598465124	0.123175258	0.039960867
A49C2N3	-51.72531003	0.986608006	0.432034035	30.26555213	58.77185886	9.710279534	0.640960342	0.123887707	0.05542739
A49C2N4	-51.72527198	0.986619992	2.80031008	17.92545093	53.06683503	24.12425377	1.817274573	0.215427679	0.050447938
A49C2N5	-51.72526301	0.986619992	5.895628264	20.40866924	46.1314055	23.73660833	3.434693336	0.368501709	0.024493616
A49C2N6	-51.72527701	0.986637007	1.602896947	18.88332927	59.02879573	18.87014862	1.355310404	0.189721489	0.069797537
A49C2N7	-51.72528304	0.98663399	0.869167657	15.24002449	58.58547987	23.33520466	1.79926131	0.137158276	0.033703742
A49C2N8	-51.72529201	0.986605994	0.94601084	19.27948228	59.58335788	18.47733737	1.465964875	0.2041179	0.043728853
A49C2N9	-51.72526804	0.98665704	3.042886215	22.37480298	52.70959279	19.66790367	1.977215139	0.189092396	0.038506814
A58N1	-51.75169299	0.977032017	0	0.143299729	1.727317102	5.864181543	68.92796661	22.53227606	0.804958958
A58N2	-51.75168101	0.97700201	0	0.0016465	0.141648925	5.209676832	72.0937647	22.00477884	0.548484197
A58N3	-51.75167497	0.976990024	0.065509695	0.256696144	0.700134864	6.214932715	73.25062401	18.87677836	0.635324206
A58N4	-51.75175401	0.977006033	0	0.195606133	1.870906924	5.922862064	71.1233459	20.3282976	0.558981374
A58N5	-51.75171898	0.976998992	0.527910541	1.385419277	2.674193312	5.766533587	65.8417488	22.91332623	0.890868254
A58N6	-51.75171303	0.977003016	0	0.543698775	1.30499728	5.786229303	69.58788294	22.04760144	0.729590257
A58N7	-51.75167103	0.97697502	0	0.003043091	0.526504571	9.407789614	71.4343084	18.09401753	0.534336788
A5N1	-51.61623901	0.936960988	31.14609905	34.66411745	14.57141716	7.270857565	8.995971506	3.326976932	0.024560335
A5N2	-51.61619199	0.93698102	1.049984931	1.324219459	1.122036361	6.105710026	58.61064485	31.48896974	0.298434628
A5N3	-51.61619903	0.937041035	52.34602655	27.81092132	15.51961823	3.014236294	1.021794486	0.277912916	0.009490197
A5N4	-51.61620598	0.93704598	60.64140743	27.1760413	9.244613022	1.732193071	0.905246379	0.289348843	0.011149955
A5N5	-51.61620196	0.937055033	37.94315674	37.5231622	14.80450754	5.174782728	3.592903292	0.898838315	0.062649186
A5N6	-51.61621403	0.937074982	36.62235572	31.00427322	17.75915209	7.530462839	5.432325258	1.537868148	0.113562727
A5N7	-51.61620498	0.937087974	27.03783917	38.76632298	16.05558451	7.484757894	8.056299438	2.376081126	0.223114881
A5N8	-51.61622199	0.937007004	23.62146495	41.30662532	16.60760407	5.128901426	9.894308597	3.227558426	0.213537217
A66N1	-51.75394001	0.981767038	5.020031207	40.24076383	34.81776243	10.20772956	8.039936627	1.581731619	0.092044721
A66N2	-51.75394898	0.98176402	7.120525051	53.37212445	28.88580173	4.425508192	4.838484484	1.287297264	0.070258826
A66N3	-51.75395502	0.981776007	1.360524188	33.2412444	51.26146537	11.92957592	1.760851556	0.372431208	0.073907359

Nest_ID	Long	Lat	Gravel	Very coarse sand	Coarse sand	Medium sand	Fine sand	Very fine sand	Coarse silt
A66N4	-51.75406197	0.981853036	69.83677082	5.52165798	14.28049422	8.310516324	1.764925818	0.233769307	0.051865537
A66N5	-51.75407203	0.981869968	20.66879027	26.57309633	17.56879871	14.63878526	16.93421373	3.409097149	0.207218554
A66N6	-51.75404403	0.981891006	47.54416255	8.209983673	18.46483892	20.41602215	4.386431213	0.867053898	0.111507594
A71N1	-51.77709804	0.996290026	0	0.054605459	0.105720159	29.57207272	65.39908861	4.742496611	0.126016434
A74N1	-51.78644897	0.994147025	36.91123814	13.37412516	8.750802695	18.506702	18.84236078	3.41882209	0.195949131
A741N1	-51.78764499	0.994561007	49.23913353	14.92214233	9.072777079	13.07404224	11.97540922	1.612129962	0.104365647
A741N10	-51.787402	0.994395968	1.967884092	17.95522696	31.16253121	36.77221099	11.4711649	0.610700279	0.060281577
A741N11	-51.78746201	0.994308963	0.027202669	1.889688694	13.86220104	65.69195419	17.82068758	0.686593369	0.021672456
A741N12	-51.787432	0.994349029	0.33890903	4.18865238	18.89195991	61.38894871	14.64373173	0.398734101	0.149064136
A741N13	-51.78742597	0.994379958	1.312727817	16.38797623	37.54855944	33.86268537	9.96299995	0.870474859	0.054576322
A741N14	-51.78772101	0.994720012	64.92913644	9.009559782	3.58081925	8.633746971	11.65659567	2.080617297	0.10952459
A741N2	-51.78763099	0.994593026	39.90818643	18.40161008	9.251070128	17.22860842	13.77517742	1.390567716	0.044779811
A741N3	-51.787619	0.994605012	56.41148679	14.60786313	5.891450085	9.437486629	11.50998586	2.019765794	0.121961704
A741N4	-51.78751197	0.994805004	16.10484124	19.84411497	17.12059498	25.3230049	18.62622888	2.768499303	0.212715717
A741N5	-51.78744298	0.99478296	6.430804594	5.892441143	21.21071115	35.9114334	27.58025525	2.823474903	0.150879565
A741N6	-51.78746503	0.994786983	15.46587264	6.051975952	8.125618358	32.35566336	34.20375483	3.606271742	0.190843121
A741N7	-51.787489	0.994756976	32.53082183	16.82041833	11.03360622	21.15462891	16.4069499	1.961892467	0.091682346
A741N8	-51.78750903	0.99478296	39.24225232	19.61688283	14.66477498	14.99196015	10.33603625	1.073261223	0.074832251
A741N9	-51.78752798	0.994781032	27.89853284	22.02630168	17.80481907	17.1516398	13.12611097	1.82959999	0.16299565
A74N2	-51.78652198	0.994135961	27.10656937	11.40150429	7.634941252	21.48335033	26.3857359	5.670985418	0.316913435
A83N1	-51.82424197	1.006710995	0.191793207	0.145990354	4.423268248	56.76066064	35.80989721	2.420665768	0.247724576
A83N10	-51.82423602	1.006533969	31.13454543	4.278541738	1.427111832	25.00638573	33.72760254	4.234639815	0.19117292
A83N11	-51.82415597	1.006753994	3.704276668	0.973015755	1.627149026	31.15751348	50.56053952	11.39737898	0.580126585
A83N12	-51.824405	1.006507985	27.61651511	7.627651021	3.634901849	32.22253849	24.33142075	4.196291691	0.370681088
A83N2	-51.82418497	1.006531036	0.817168371	0.330859621	0.424877649	39.19379042	50.63250079	8.21020914	0.390594005
A83N3	-51.824232	1.006573029	2.890509973	1.703302378	1.795904071	48.65445648	41.02604273	3.769477664	0.160306703

Nest_ID	Long	Lat	Gravel	Very coarse sand	Coarse sand	Medium sand	Fine sand	Very fine sand	Coarse silt
A83N4	-51.82422403	1.006568	7.356792091	3.76602512	2.528017553	37.71550377	41.2794695	7.068428682	0.285763288
A83N5	-51.82441598	1.006597001	30.74155389	4.430610183	3.37684775	30.57846211	26.72046867	3.911659117	0.240398283
A83N6	-51.82399596	1.006287038	4.762052095	0.702036645	1.029151388	34.62106589	52.32079302	6.283547351	0.281353611
A83N7	-51.82415899	1.00687	20.69449757	3.081761823	2.249371512	21.94907577	44.45113078	7.172949213	0.401213328
A83N8	-51.82422998	1.006817026	18.54630965	4.685489036	5.642770059	34.23808612	30.97549879	5.563404029	0.348442311
A83N9	-51.82425102	1.006627008	1.419744985	0.256602064	0.382557647	34.31825543	57.11292237	6.141482453	0.368435052
A87N1	-51.81566603	1.001749998	43.72114333	4.513306446	4.455062828	18.25695367	24.40926647	4.413339036	0.23092821
A87N2	-51.815691	1.001758967	21.54998988	1.608546867	1.622061553	25.84112713	42.12188551	6.900419205	0.355969851
A87N3	-51.81569796	1.001765002	16.64681008	5.114806017	5.332753871	26.96009451	39.27097617	6.426289616	0.24826973
A87N4	-51.81568002	1.001760978	22.89125627	4.373384127	4.686124872	24.38315728	36.48321237	6.845541874	0.337323204
A87N5	-51.81566401	1.001759972	22.81939774	5.355248251	5.406498948	29.79653573	31.34654462	5.004635541	0.271139164
A9N1	-51.62638304	0.947117005	0.631937669	20.64951176	68.7494002	9.194810544	0.628388861	0.124708098	0.021242862
A9N2	-51.62638497	0.947112981	1.115257976	40.12950738	54.37581916	4.203195927	0.128418499	0.038210872	0.00959018
A9N3	-51.62640299	0.947129996	0.707690955	31.17847884	60.68887183	6.522579949	0.620291472	0.246307634	0.03577932
A9N4	-51.62641698	0.947115999	0.543572804	18.18829394	70.25461042	9.772363267	0.976871412	0.214349842	0.049938314
A9N5	-51.62643199	0.947142988	0.160588836	16.28467576	74.35557431	7.73944399	0.889000638	0.495036663	0.075679796
BF10N1	-51.60054096	0.953263035	8.447389433	49.69521084	30.88340826	7.059958675	2.768574065	1.056740798	0.088717923
BF10N10	-51.60062796	0.953083998	1.779762609	12.02497135	36.8267585	27.26440071	17.60226925	4.374376726	0.127460855
BF10N11	-51.60061799	0.953093972	9.896971745	25.91249711	31.53320479	15.53135596	14.7757522	2.288124722	0.062093479
BF10N12	-51.600491	0.95323001	10.09154439	46.59779417	33.04776769	6.781501855	2.888282508	0.562562983	0.030546406
BF10N2	-51.60051196	0.953269992	14.60315651	57.97754753	20.66240339	3.250812928	2.656574006	0.819053648	0.030451995
BF10N3	-51.60052403	0.953290025	2.072084193	39.49606841	50.26385027	6.383681646	1.29877119	0.473335924	0.012208369
BF10N4	-51.60051598	0.953311985	10.66211656	47.69505955	30.06832154	7.490494524	3.220722836	0.816082286	0.047202703
BF10N5	-51.60048899	0.953292036	5.93851219	35.3494355	40.60600684	11.1919437	5.849799574	1.028219084	0.036083109
BF10N6	-51.60049201	0.953292036	7.098185213	40.38388083	38.38663737	9.005422322	4.113664311	0.962594257	0.04961569
BF10N7	-51.60049402	0.953327995	8.001660386	42.44526852	36.73038982	9.029449347	3.338126114	0.44675387	0.008351942

Nest_ID	Long	Lat	Gravel	Very coarse sand	Coarse sand	Medium sand	Fine sand	Very fine sand	Coarse silt
BF10N8	-51.60045597	0.953302011	2.024922895	15.70911714	45.82797435	22.65695547	11.92917459	1.794831627	0.05702392
BF10N9	-51.60048103	0.953325983	10.55282877	37.25649267	35.76689294	11.4656829	4.332740105	0.610919488	0.01444312
BF18N1	-51.60869999	0.963155022	19.16633235	17.29552499	20.06813006	23.93877975	16.7000481	2.672261176	0.158923571
BF24N1	-51.60458196	0.969652003	0.027688076	5.256493852	58.69697422	32.38686677	3.203385628	0.38853105	0.040060405
BF24N2	-51.60457802	0.969639011	0.136411013	4.4878924	57.32959725	34.99606085	2.800566999	0.229364092	0.020107402
BF27N1	-51.60058396	0.974930003	0	0.040271689	0.129009306	10.78716662	67.62550927	20.55479998	0.86324314
BF27N2	-51.60053501	0.974977026	0	0.012331971	0.098256349	11.10736104	70.41275655	17.85604446	0.513249629
BF27N3	-51.60047801	0.974980965	0	0.016119446	0.091925755	11.51402517	70.50840133	17.42681747	0.442710842
BF27N4	-51.60045303	0.974966967	0.015382971	0.042752673	0.109878365	10.4199651	68.47599707	20.12751884	0.808504985
BF27N5	-51.60047399	0.974963028	0.055146875	0.056095102	0.108097857	12.71861619	71.49510416	15.11009412	0.456845699
BF27N6	-51.60045697	0.975006027	0.228418631	0.150166411	0.170627769	13.20241724	70.2256289	15.48665345	0.536087599
BF27N7	-51.60046804	0.974991024	0.066811208	0.070006961	0.092327297	12.19709007	68.7984169	18.14039142	0.634956143

Supplementary Table S2. *Podocnemis unifilis* nests sampled in the study area with environmental and anthropogenic variables.

Nest_ID	Type	River	Dist_house	Nest_density	Dist_water	PC1
2012P041N1	Bank	Falsino	10.61331208	0.008381199	3.2	0.226600156
2012P041N2	Bank	Falsino	10.61331208	0.008381199	3.5	0.101025471
2012P041N3	Bank	Falsino	10.61331208	0.008381199	2.7	1.334835077
2012P041N4	Bank	Falsino	10.61331208	0.008381199	3.5	0.782457918
2012P041N5	Bank	Falsino	10.61331208	0.008381199	2.3	0.356155614
2012P041N6	Bank	Falsino	10.61331208	0.008381199	3.1	0.316481583
2012P041N7	Bank	Falsino	10.61331208	0.008381199	1.3	0.341425627
2012P041N8	Bank	Falsino	10.61331208	0.008381199	6.5	0.619265096
2012P041N9	Bank	Falsino	10.61331208	0.008381199	5.6	0.74030423
2012P05N1	Island	Falsino	13.734414	0.010160559	2.2	-0.353941224
2012P05N2	Island	Falsino	13.734414	0.010160559	1.7	-0.456394413
2012P06N1	Island	Falsino	15.27258715	0.015634465	5	-0.27678132
2012P06N2	Island	Falsino	15.27258715	0.015634465	4.8	-0.005010019
2012P06N3	Island	Falsino	15.27258715	0.015634465	4.45	0.115398233
2012P06N4	Island	Falsino	15.27258715	0.015634465	4	-0.699363537
2012P07N1	Island	Falsino	15.35055527	0.008417599	2.4	1.403953701
2012P07N10	Island	Falsino	15.35055527	0.008417599	12	1.150707449
2012P07N11	Island	Falsino	15.35055527	0.008417599	3.9	1.497669304
2012P07N2	Island	Falsino	15.35055527	0.008417599	3.8	0.133040065
2012P07N3	Island	Falsino	15.35055527	0.008417599	2.9	1.761207355
2012P07N4	Island	Falsino	15.35055527	0.008417599	2.4	0.965845606
2012P07N5	Island	Falsino	15.35055527	0.008417599	2.4	1.493169159
2012P07N6	Island	Falsino	15.35055527	0.008417599	2.3	1.334202092
2012P07N7	Island	Falsino	15.35055527	0.008417599	13.8	1.343534878

Nest_ID	Type	River	Dist_house	Nest_density	Dist_water	PC1
2012P07N8	Island	Falsino	15.35055527	0.008417599	13.8	1.388987474
2012P07N9	Island	Falsino	15.35055527	0.008417599	12.8	1.410366845
2012P081N1	Island	Falsino	1.77915644	0.101239331	4.65	-2.190747199
2012P081N2	Island	Falsino	1.77915644	0.101239331	4.1	-1.947138938
2012P081N3	Island	Falsino	1.77915644	0.101239331	3.75	-1.047842423
2012P081N4	Island	Falsino	1.77915644	0.101239331	3.95	-2.265271399
2012P081N5	Island	Falsino	1.77915644	0.101239331	5.45	-1.823321203
2012P091N1	Island	Falsino	3.0710498	0.003151018	6.7	-1.554974569
2012P12N1	Island	Falsino	13.30040956	0.021808536	8	1.46546903
2012P12N2	Island	Falsino	13.30040956	0.021808536	4.5	1.719043678
2012P13N1	Island	Falsino	16.15592999	0.005414502	3.5	0.064664118
2012P13N2	Island	Falsino	16.15592999	0.005414502	13.6	0.73649141
2012P13N3	Island	Falsino	16.15592999	0.005414502	6	0.860665463
2012P13N4	Island	Falsino	16.15592999	0.005414502	1.7	0.097272752
2012P13N5	Island	Falsino	16.15592999	0.005414502	1.6	0.45792513
2012P16N1	Bank	Falsino	15.5061197	0.005124947	5.3	-1.606513653
2012P16N2	Bank	Falsino	15.5061197	0.005124947	3	-1.117880959
2012P16N3	Bank	Falsino	15.5061197	0.005124947	7	-2.773220532
2012P17N1	Island	Falsino	13.34136272	0.007704013	6.8	0.519180445
2012P241N1	Island	Falsino	7.14559296	0.051051479	4.85	0.435257812
2012P242N1	Island	Falsino	7.13230171	0.065257589	2.65	1.388438323
2012P242N2	Island	Falsino	7.13230171	0.065257589	2.2	1.627649325
2012P242N3	Island	Falsino	7.13230171	0.065257589	2.9	1.666330096
2012P242N4	Island	Falsino	7.13230171	0.065257589	2.3	-0.082388467
2012P28N1	Bank	Falsino	19.04516364	0.003260367	3.2	1.507671846
2012P28N2	Bank	Falsino	19.04516364	0.003260367	4.2	1.971652829

Nest_ID	Type	River	Dist_house	Nest_density	Dist_water	PC1
2012P28N3	Bank	Falsino	19.04516364	0.003260367	1.5	1.593540756
2012P28N4	Bank	Falsino	19.04516364	0.003260367	4.65	2.20799986
2012P28N5	Bank	Falsino	19.04516364	0.003260367	6.1	1.404568053
2012P28N6	Bank	Falsino	19.04516364	0.003260367	6.6	-1.100567163
2017A1N1	Island	Araguari	1.02092114	0.077019661	2.6	-4.55579885
2017A1N2	Island	Araguari	1.02092114	0.077019661	4.15	-3.89343915
2017A1N3	Island	Araguari	1.02092114	0.077019661	4.5	-4.319953474
2017A1N4	Island	Araguari	1.02092114	0.077019661	3	-4.134095854
2017A1N5	Island	Araguari	1.02092114	0.077019661	1.8	-2.857523342
2017A1N6	Island	Araguari	1.02092114	0.077019661	3.2	-4.419119758
2017A2N1	Bank	Araguari	0.52364669	0.01492796	3.8	-4.65257671
2017A3N1	Island	Araguari	0.57616081	0.016100374	3.5	0.233339718
2017A3N2	Island	Araguari	0.57616081	0.016100374	4.7	-0.167573491
2017A3N3	Island	Araguari	0.57616081	0.016100374	5	0.233339718
2017A4N1	Bank	Araguari	6.13204853	0.02405714	5.4	-2.065340407
2017A4N2	Bank	Araguari	6.13204853	0.02405714	4.1	-1.619170874
2017A4N3	Bank	Araguari	6.13204853	0.02405714	4.4	-2.216733354
2017A4N4	Bank	Araguari	6.13204853	0.02405714	4.4	-1.478053534
2017A5N1	Bank	Araguari	11.02073505	0.020444421	4.8	-2.700987999
2017A5N2	Bank	Araguari	11.02073505	0.020444421	3.8	-2.953465884
2017A6N1	Bank	Araguari	2.34809123	0.050130148	8	-4.332691964
2017A6N2	Bank	Araguari	2.34809123	0.050130148	7.5	-4.240280724
2017A6N3	Bank	Araguari	2.34809123	0.050130148	7.7	-4.099071629
2017A6N4	Bank	Araguari	2.34809123	0.050130148	8.2	-4.370364163
2017A6N5	Bank	Araguari	2.34809123	0.050130148	8.2	-4.659954927
2017P2N1	Bank	Falsino	1.72551371	0.011474231	5	1.577000111

Nest_ID	Type	River	Dist_house	Nest_density	Dist_water	PC1
2017P3N1	Bank	Falsino	2.90104202	0.016372528	2.9	-0.88411262
2017P3N2	Bank	Falsino	2.90104202	0.016372528	2.9	-0.892405594
2017P3N3	Bank	Falsino	2.90104202	0.016372528	2.9	-0.955402401
2017P3N4	Bank	Falsino	2.90104202	0.016372528	2.6	-0.640665343
2017P3N5	Bank	Falsino	2.90104202	0.016372528	2.4	-1.03905832
2017P3N6	Bank	Falsino	2.90104202	0.016372528	3.16	-0.919252305
2017P4N1	Bank	Falsino	8.62390203	0.015924949	2.35	-1.152926559
2017P4N2	Bank	Falsino	8.62390203	0.015924949	2.25	-1.276482044
2017P5N1	Bank	Falsino	12.24137042	0.008229929	2.53	0.610358233
2017P5N2	Bank	Falsino	12.24137042	0.008229929	2.73	0.865294431
2017P5N3	Bank	Falsino	12.24137042	0.008229929	5.4	1.630122313
2017P5N4	Bank	Falsino	12.24137042	0.008229929	7.05	1.2735391
2017P6N1	Island	Falsino	13.35959946	0.014705863	2.35	1.658750084
2017P6N2	Island	Falsino	13.35959946	0.014705863	2.35	1.574452568
2017P6N3	Island	Falsino	13.35959946	0.014705863	2.4	1.262798156
2017P6N4	Island	Falsino	13.35959946	0.014705863	2.35	1.86908581
2017P6N5	Island	Falsino	13.35959946	0.014705863	3.5	1.256168283
2017P6N6	Island	Falsino	13.35959946	0.014705863	3	1.90443992
A181N1	Island	Araguari	0.77069358	0.012249973	8.7	1.240011287
A181N10	Island	Araguari	0.77069358	0.012249973	5.3	-0.398123067
A181N11	Island	Araguari	0.77069358	0.012249973	6.6	0.878907273
A181N12	Island	Araguari	0.77069358	0.012249973	6.1	0.648421834
A181N13	Island	Araguari	0.77069358	0.012249973	3.85	0.116344817
A181N2	Island	Araguari	0.77069358	0.012249973	6.7	1.098647055
A181N3	Island	Araguari	0.77069358	0.012249973	1.3	-1.221409941
A181N4	Island	Araguari	0.77069358	0.012249973	1.8	0.016359817

Nest_ID	Type	River	Dist_house	Nest_density	Dist_water	PC1
A181N5	Island	Araguari	0.77069358	0.012249973	3.6	0.081983849
A181N6	Island	Araguari	0.77069358	0.012249973	3.6	0.709331947
A181N7	Island	Araguari	0.77069358	0.012249973	3.3	0.118259808
A181N8	Island	Araguari	0.77069358	0.012249973	4.4	0.756535633
A181N9	Island	Araguari	0.77069358	0.012249973	5.6	0.097866986
A19N1	Bank	Araguari	0.10803818	0.002237391	17.6	1.166316606
A19N2	Bank	Araguari	0.10803818	0.002237391	16.8	1.544299168
A19N3	Bank	Araguari	0.10803818	0.002237391	16.1	1.3193316
A19N4	Bank	Araguari	0.10803818	0.002237391	16.4	0.271762772
A37N1	Island	Araguari	0.06158254	0.015323629	6.6	-0.726811091
A37N2	Island	Araguari	0.06158254	0.015323629	5.4	-0.392596921
A37N3	Island	Araguari	0.06158254	0.015323629	5.95	-0.790103778
A37N4	Island	Araguari	0.06158254	0.015323629	6.6	0.90112617
A40C2N1	Bank	Araguari	0.92485816	0.008635034	3.8	1.529023768
A40C2N2	Bank	Araguari	0.92485816	0.008635034	3.5	1.664500746
A49C2N1	Island	Araguari	3.10192911	0.052098315	3.9	2.161867167
A49C2N10	Island	Araguari	3.10192911	0.052098315	3.5	1.91513722
A49C2N11	Island	Araguari	3.10192911	0.052098315	3.8	1.412059955
A49C2N12	Island	Araguari	3.10192911	0.052098315	3.4	0.026857464
A49C2N13	Island	Araguari	3.10192911	0.052098315	2.2	0.767079512
A49C2N14	Island	Araguari	3.10192911	0.052098315	1.9	0.517447912
A49C2N15	Island	Araguari	3.10192911	0.052098315	3.6	0.40969046
A49C2N16	Island	Araguari	3.10192911	0.052098315	2.9	0.64047477
A49C2N17	Island	Araguari	3.10192911	0.052098315	2.1	1.58023945
A49C2N2	Island	Araguari	3.10192911	0.052098315	3.9	2.249592879
A49C2N3	Island	Araguari	3.10192911	0.052098315	3.1	2.221443569

Nest_ID	Type	River	Dist_house	Nest_density	Dist_water	PC1
A49C2N4	Island	Araguari	3.10192911	0.052098315	4	1.815104911
A49C2N5	Island	Araguari	3.10192911	0.052098315	4.1	1.772510833
A49C2N6	Island	Araguari	3.10192911	0.052098315	3.6	1.917484567
A49C2N7	Island	Araguari	3.10192911	0.052098315	4.2	1.86362627
A49C2N8	Island	Araguari	3.10192911	0.052098315	3.6	1.967928628
A49C2N9	Island	Araguari	3.10192911	0.052098315	4	1.935991307
A58N1	Bank	Araguari	5.863997	0.053421787	7.2	-3.777111277
A58N2	Bank	Araguari	5.863997	0.053421787	5.3	-3.445355666
A58N3	Bank	Araguari	5.863997	0.053421787	3.7	-3.369449795
A58N4	Bank	Araguari	5.863997	0.053421787	7	-3.286964241
A58N5	Bank	Araguari	5.863997	0.053421787	7.8	-3.816456001
A58N6	Bank	Araguari	5.863997	0.053421787	8.8	-3.639084264
A58N7	Bank	Araguari	5.863997	0.053421787	3.7	-3.128594418
A5N1	Bank	Araguari	2.21838274	0.025408989	2	1.453769132
A5N2	Bank	Araguari	2.21838274	0.025408989	6	-3.365641242
A5N3	Bank	Araguari	2.21838274	0.025408989	4.6	1.914197415
A5N4	Bank	Araguari	2.21838274	0.025408989	4	1.861647484
A5N5	Bank	Araguari	2.21838274	0.025408989	6.8	1.820721925
A5N6	Bank	Araguari	2.21838274	0.025408989	5.9	1.539510283
A5N7	Bank	Araguari	2.21838274	0.025408989	7	1.322150693
A5N8	Bank	Araguari	2.21838274	0.025408989	3.6	1.276952571
A66N1	Island	Araguari	6.14360572	0.007999225	3.1	1.751212979
A66N2	Island	Araguari	6.14360572	0.007999225	2.4	2.107404226
A66N3	Island	Araguari	6.14360572	0.007999225	3	2.095648793
A66N4	Island	Araguari	6.14360572	0.007999225	5.1	1.4340773
A66N5	Island	Araguari	6.14360572	0.007999225	3	0.750577336

Nest_ID	Type	River	Dist_house	Nest_density	Dist_water	PC1
A66N6	Island	Araguari	6.14360572	0.007999225	5.4	1.172737396
A71N1	Bank	Araguari	8.93684121	0.010458538	2.9	-1.437609896
A74N1	Island	Araguari	9.9044173	0.003391249	13.9	0.396048997
A741N1	Island	Araguari	10.0404001	0.006883422	4	0.973732467
A741N10	Island	Araguari	10.0404001	0.006883422	12	1.155435282
A741N11	Island	Araguari	10.0404001	0.006883422	12.6	0.347017528
A741N12	Island	Araguari	10.0404001	0.006883422	11.5	0.390105423
A741N13	Island	Araguari	10.0404001	0.006883422	12	1.250895711
A741N14	Island	Araguari	10.0404001	0.006883422	5	0.843227978
A741N2	Island	Araguari	10.0404001	0.006883422	6.5	1.042437183
A741N3	Island	Araguari	10.0404001	0.006883422	7.6	0.929967995
A741N4	Island	Araguari	10.0404001	0.006883422	8.6	0.535035507
A741N5	Island	Araguari	10.0404001	0.006883422	6.7	0.074257005
A741N6	Island	Araguari	10.0404001	0.006883422	7.6	-0.336884658
A741N7	Island	Araguari	10.0404001	0.006883422	11.1	0.796978264
A741N8	Island	Araguari	10.0404001	0.006883422	11.4	1.215325142
A741N9	Island	Araguari	10.0404001	0.006883422	13	0.974305365
A74N2	Island	Araguari	9.9044173	0.003391249	13	-0.274203256
A83N1	Island	Araguari	14.32457723	0.00447443	19.6	-0.731215551
A83N10	Island	Araguari	14.32457723	0.00447443	6	-0.388992558
A83N11	Island	Araguari	14.32457723	0.00447443	18.9	-2.194547532
A83N12	Island	Araguari	14.32457723	0.00447443	8.6	-0.368871236
A83N2	Island	Araguari	14.32457723	0.00447443	6.25	-1.744695153
A83N3	Island	Araguari	14.32457723	0.00447443	9.95	-0.789409655
A83N4	Island	Araguari	14.32457723	0.00447443	9	-1.114300413
A83N5	Island	Araguari	14.32457723	0.00447443	16.4	-0.253213705

Nest_ID	Type	River	Dist_house	Nest_density	Dist_water	PC1
A83N6	Island	Araguari	14.29097595	0.001884028	11	-1.421216868
A83N7	Island	Araguari	14.32457723	0.00447443	5.6	-1.266691672
A83N8	Island	Araguari	14.32457723	0.00447443	9.5	-0.696037077
A83N9	Island	Araguari	14.32457723	0.00447443	19.4	-1.705087786
A87N1	Island	Araguari	13.27145774	0.004954554	3	-0.079788542
A87N2	Island	Araguari	13.27145774	0.004954554	5.3	-1.165752178
A87N3	Island	Araguari	13.27145774	0.004954554	5.4	-0.796594782
A87N4	Island	Araguari	13.27145774	0.004954554	4.5	-0.873736419
A87N5	Island	Araguari	13.27145774	0.004954554	2.2	-0.493561476
A9N1	Island	Araguari	3.54246854	0.026124738	3.7	2.223492414
A9N2	Island	Araguari	3.54246854	0.026124738	3	2.476265363
A9N3	Island	Araguari	3.54246854	0.026124738	2.6	2.303337629
A9N4	Island	Araguari	3.54246854	0.026124738	1.3	2.131784621
A9N5	Island	Araguari	3.54246854	0.026124738	4	2.098822085
BF10N1	Bank	Falsino	1.88839116	0.032379828	2	2.099623927
BF10N10	Bank	Falsino	1.88839116	0.032379828	1.8	0.610510273
BF10N11	Bank	Falsino	1.88839116	0.032379828	2.4	1.237438984
BF10N12	Bank	Falsino	1.88839116	0.032379828	2.2	2.200796599
BF10N2	Bank	Falsino	1.88839116	0.032379828	2.9	2.282455585
BF10N3	Bank	Falsino	1.88839116	0.032379828	2.6	2.334968387
BF10N4	Bank	Falsino	1.88839116	0.032379828	3.4	2.127964756
BF10N5	Bank	Falsino	1.88839116	0.032379828	2	1.91918942
BF10N6	Bank	Falsino	1.88839116	0.032379828	2	2.034087062
BF10N7	Bank	Falsino	1.88839116	0.032379828	4	2.179559837
BF10N8	Bank	Falsino	1.88839116	0.032379828	4.4	1.279782221
BF10N9	Bank	Falsino	1.88839116	0.032379828	1.9	2.016897316

Nest_ID	Type	River	Dist_house	Nest_density	Dist_water	PC1
BF18N1	Island	Falsino	1.62845977	0.016641877	2	0.687401172
BF24N1	Island	Falsino	0.84642766	0.369269924	1.5	1.556510509
BF24N2	Island	Falsino	0.84642766	0.369269924	1.2	1.564823603
BF27N1	Bank	Falsino	0.3985577	0.013169389	4.5	-3.731941063
BF27N2	Bank	Falsino	0.3985577	0.013169389	3	-3.0644586
BF27N3	Bank	Falsino	0.3985577	0.013169389	2.5	-2.92748465
BF27N4	Bank	Falsino	0.3985577	0.013169389	1.9	-3.636239746
BF27N5	Bank	Falsino	0.3985577	0.013169389	2.6	-2.807288048
BF27N6	Bank	Falsino	0.3985577	0.013169389	4.8	-2.923233541
BF27N7	Bank	Falsino	0.3985577	0.013169389	3	-3.235183406

Supplementary Figure S2. Tracks of adults of *Podocnemis unifilis* in nesting areas during nesting season obtained in three different substrate types in our study area. (A) fine sand, (B) medium sand, (C) very coarse sand and gravel. Photo credits: Fernanda Michalski.

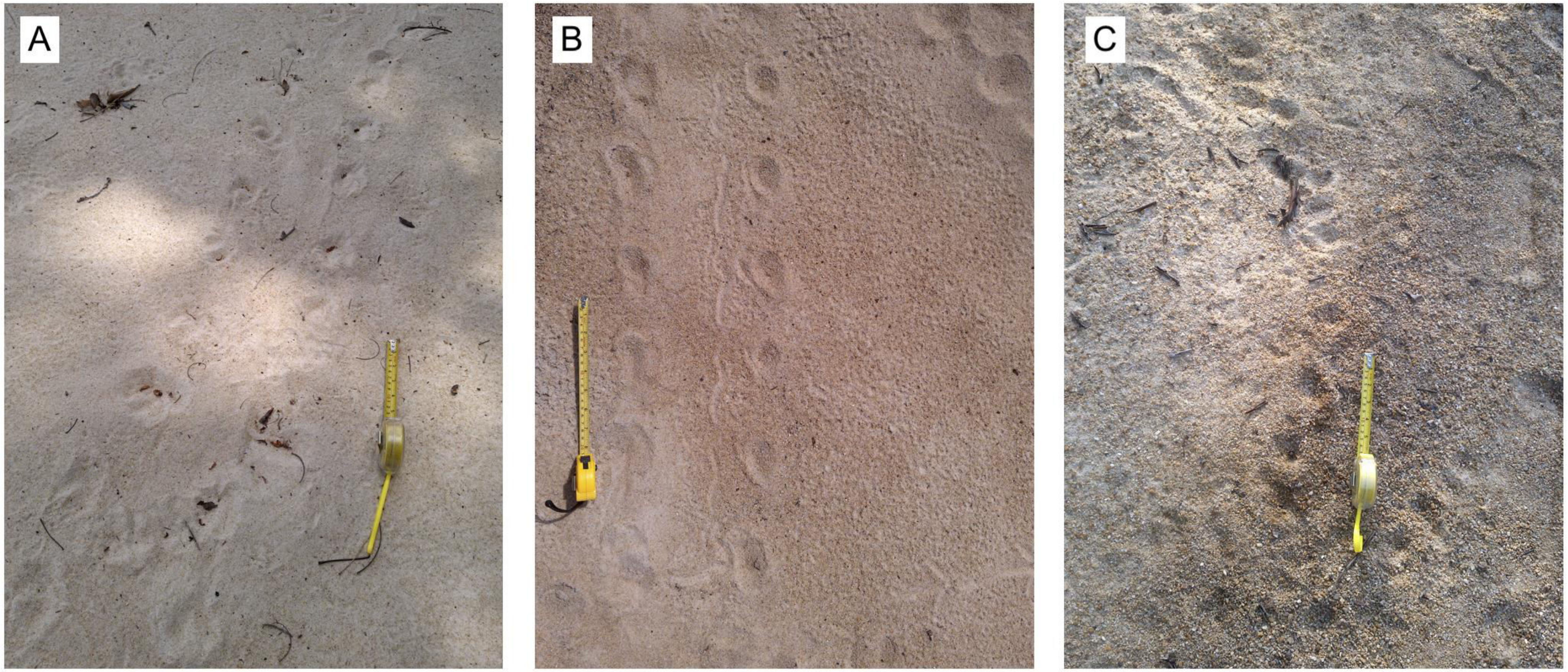

Supplementary Table S3. Importance of components.

	PC1	PC2	PC3	PC4	PC5	PC6	PC7
Eigenvalue	1.86	1.30	1.06	0.64	0.51	0.24	1.05e-10
Proportion of variance	0.49	0.24	0.16	0.06	0.04	0.01	0.00e+00
Cumulative proportion	0.49	0.73	0.90	0.95	0.99	1.00	1.00e+00

Supplementary Table S4. Three first principal components (PC) after varimax rotation.

Grain size substrate	PC1	PC2	PC3
Gravel	0.17181796	0.46599075	0.642973954
Very coarse sand	0.34516885	0.43234559	-0.255065114
Coarse sand	0.36235656	-0.12522251	-0.604269608
Medium sand	-0.00345146	-0.71417979	0.261324243
Fine sand	-0.50925490	0.01344032	0.007177055
Very fine sand	-0.49706366	0.18930901	-0.205751566
Coarse silt	-0.46217965	0.18487270	-0.213796504
